# Systems level analysis of sex-dependent gene expression changes in Parkinson’s disease

**DOI:** 10.1038/s41531-023-00446-8

**Published:** 2023-01-21

**Authors:** Léon-Charles Tranchevent, Rashi Halder, Enrico Glaab

**Affiliations:** Luxembourg Centre for Systems Biomedicine (LCSB), University of Luxembourg, Esch-sur-Alzette, Luxembourg

**Keywords:** Systems biology, High-throughput screening

## Abstract

Parkinson’s disease (PD) is a heterogeneous disorder, and among the factors which influence the symptom profile, biological sex has been reported to play a significant role. While males have a higher age-adjusted disease incidence and are more frequently affected by muscle rigidity, females present more often with disabling tremors. The molecular mechanisms involved in these differences are still largely unknown, and an improved understanding of the relevant factors may open new avenues for pharmacological disease modification. To help address this challenge, we conducted a meta-analysis of disease-associated molecular sex differences in brain transcriptomics data from case/control studies. Both sex-specific (alteration in only one sex) and sex-dimorphic changes (changes in both sexes, but with opposite direction) were identified. Using further systems level pathway and network analyses, coordinated sex-related alterations were studied. These analyses revealed significant disease-associated sex differences in mitochondrial pathways and highlight specific regulatory factors whose activity changes can explain downstream network alterations, propagated through gene regulatory cascades. Single-cell expression data analyses confirmed the main pathway-level changes observed in bulk transcriptomics data. Overall, our analyses revealed significant sex disparities in PD-associated transcriptomic changes, resulting in coordinated modulations of molecular processes. Among the regulatory factors involved, NR4A2 has already been reported to harbor rare mutations in familial PD and its pharmacological activation confers neuroprotective effects in toxin-induced models of Parkinsonism. Our observations suggest that NR4A2 may warrant further research as a potential adjuvant therapeutic target to address a subset of pathological molecular features of PD that display sex-associated profiles.

## Introduction

Parkinson’s disease (PD) has a worldwide prevalence projected at 12 million by 2040 and no disease-modifying treatments are available^1^. Current therapies focus on the replacement of dopamine, but can alleviate only some of the motor symptoms, are hampered by severe adverse effects and loss of efficacy over time, and do not address many of the other heterogeneous symptoms^2–5^. There is widespread agreement in the field that PD patients with diverse clinical features and disease course have different therapeutic needs and would benefit from more personalized medical approaches^6–10^.

A striking aspect in the heterogeneity of PD are the pronounced and multifaceted sex differences observed in previous epidemiological and clinical studies. Both the incidence and prevalence of PD is approximately 1.5–2 times greater in men than in women^11–16^, but female patients present significantly more often with a phenotype dominated by disabling tremors^3,17^ and, irrespective of their body weight, have an almost 3-fold increased risk to develop treatment-related complications (e.g., involuntary muscle movements known as dyskinesias)^18,19^. Moreover, while males tend to display a lower striatal dopamine transporter binding^17,20–22^, females tend to be affected more often by motor and non-motor symptom fluctuations^23–25^.

Previous studies have suggested that potential neuroprotective functions of certain sex-related hormones may contribute to sex differences in neurologic disorders^26–29^. However, it is unclear how exactly they may influence the molecular hallmarks of PD, and why in spite of generic sex differences in hormone levels, an almost opposite association between biological sex and disease risk is observed in PD as compared to other degenerative disorders, such as Alzheimer’s disease^30–32^. Life-style and occupation related differences have been proposed as contributing factors to PD sex differences, e.g., exposure to PD-associated toxicants and head trauma are more common among males^16^, but these associations are not strong enough to explain the full extent of the reported disparities. While a variety of meta-analyses of PD omics data have previously already been conducted^33–37^, to the best of our knowledge, similar integrative analyses have not yet been applied to study molecular sex differences.

In order to contribute to a more detailed molecular-level understanding of disease-associated sex differences, we therefore present a comprehensive statistical meta-analysis of PD transcriptomics data from brain tissue samples of post-mortem case–control studies. Both sex-specific changes, i.e., significant molecular alterations occurring either only in females or only in males, and sex-dimorphic changes, i.e., alterations with opposite direction across both analyses are determined (see “Methods” for details). Finally, in order to understand the coordination and regulation of sex-related PD-associated molecular alterations, we determine pathway- and network activity changes with significant disease-related sex differences and identify transcription factors that may play a key role in regulating the observed downstream changes. The results derived from bulk transcriptomics data are then further examined and characterized in cell-type-specific analyses of corresponding single-cell transcriptomics datasets.

## Results

### Gene-level analysis of PD-associated molecular sex differences

The gene-level statistical meta-analysis, conducted independently for each biological sex on the *substantia nigra* (SN) tissue samples from twelve transcriptomics datasets (see “Methods”), identified 1146 significantly differentially expressed genes (DEGs) in males and 118 DEGs in females after multiple testing adjustments (out of 11,959 and 11,975 genes, respectively). Overall, the meta-analysis allowed us to identify more significantly differentially expressed genes than when using the individual datasets in isolation. Since males were over-represented among the samples from the available datasets, in line with the previously reported higher relative risk for males of developing PD^16^, we decided to further investigate whether the larger number of male-specific DEGs may have resulted, at least partly, from a higher statistical power of the associated analysis. We have therefore repeated the meta-analyses with three equally sized random subsets of male samples as compared to the female samples. Interestingly, we obtained similar differential expression patterns when sub-sampling male samples (see Supplementary Note 1). These results indicate that the lower number of significant DEGs in females may at least partly reflect a different disease manifestation and progression, with a distinct extent of disease-related gene expression variations in females, in line with the findings from prior studies^38–40^.

**Fig. 1:**
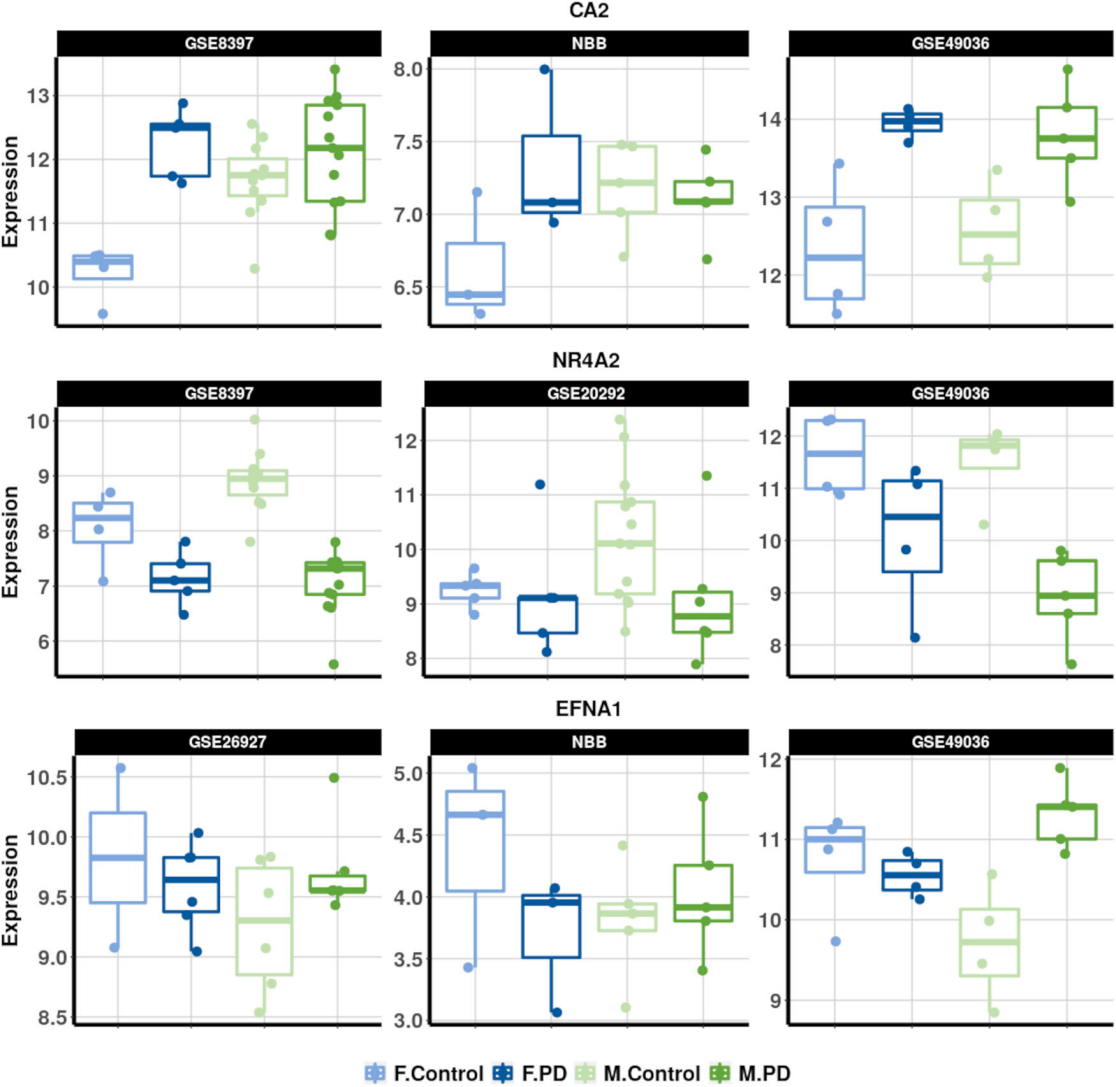
Expression levels of selected differentially expressed genes in representative datasets. The selected genes are *CA2* (top, female-specific), *NR4A2* (middle, male-specific), and *EFNA1* (bottom, candidate sex-dimorphic). Each subplot contains four boxplots corresponding to the four categories of interest: female controls (light blue), female PD patients (dark blue), male controls (light green) and male PD patients (dark green). The data used to produce the boxplots is the pre-processed transcriptomics data. Each boxplot is represented by the median, two hinges (representing the first and third quartiles) and two whiskers (extending up to 1.5 × inter-quartile range from the hinges).

Detailed differential expression statistics for the top-ranked DEGs, including the base 2 log. fold changes (LFC), false-discovery rates (FDR) and consistency scores for both males and females, are presented in Table 1. Expression profiles for selected genes in representative datasets are presented in Fig. 1. The DEGs are split into three categories, depending on whether their alterations are male- or female-specific, or whether they display sex-dimorphic alterations with an increase/decrease of expression levels in males and the opposite change in females. In total, we identified 36 female-specific genes (i.e., significantly differentially expressed between PD and controls in females only with FDR < 0.05, and not approaching significance in males, based on comparing male and female *π*-value rankings^41^) 539 male-specific genes (i.e., FDR < 0.05 in males and not approaching significance in females) and 37 candidate sex-dimorphic genes (i.e., FDR < 0.05 in at least one sex, but with opposite signs of the log. fold change, and a minimum absolute cross-study log. fold change of 0.25 to ensure the robustness of the difference). Table 1 shows the top ten genes for each of these categories, and the complete lists of significant sex-specific and candidate sex-dimorphic DEGs are provided in Supplementary Table 5.

**Table 1:** Top sex-specific and candidate sex-dimorphic genes.

	Gene	Female Cst	Female LFC	Female FDR	Male Cst	Male LFC	Male FDR
Female-specific genes	H2AC6	0.70	0.98	1.22e−04	0.86	0.45	8.70e−01
	CA2	0.81	1.10	8.17e−04	0.63	0.62	4.52e−01
	H2BC21	0.86	0.75	8.34e−04	0.72	0.43	1.77e−01
	CXCR4	0.81	0.92	1.79e−03	0.56	−0.21	1
	SLC2A6	0.81	−0.64	1.79e−03	1.00	−0.51	1.75e−01
	SALL1	0.72	1.01	2.57e−03	0.74	0.41	9.42e−01
	SGSH	0.74	−0.55	4.29e−03	0.84	−0.32	6.90e−01
	AMD1^a^	0.72	0.65	4.65e−03	0.86	0.39	1
	PRUNE2	0.62	0.61	5.57e−03	0.74	0.21	1
	POGK	0.81	0.61	6.44e−03	0.86	0.35	1.82e−01
Male-specific genes	DENR	0.86	−0.42	9.65e−01	0.86	−0.88	9.48e−11
	NR4A2	0.86	−0.64	1	0.86	−1.40	1.58e−08
	NELL2	0.89	−0.82	1	0.89	−1.30	2.09e−08
	RAB6B	0.63	−0.42	9.10e−01	0.63	−0.82	6.50e−07
	GNG3	0.86	−0.66	1	0.86	−1.23	1.72e−06
	SNCA	1.00	−0.36	1	1.00	−0.82	1.92e−06
	KIF3C	0.89	−0.33	1	0.89	−0.83	6.00e−06
	DYNC1I1	1.00	−0.50	9.31e−01	1.00	−0.94	6.35e−06
	REEP1	0.86	−0.58	1	0.86	−1.22	8.22e−06
	DMXL2	1.00	−0.53	9.56e−01	1.00	−1.00	8.46e−06
Sex-dimorphic genes	EFNA1	0.62	−0.32	1	0.89	0.76	9.33e−05
	ARMCX2	0.60	0.34	1	0.72	−0.61	3.69e−04
	SH3TC1	0.62	−0.34	1	0.73	0.80	7.54e−04
	RUSC1	0.60	0.25	1	0.74	−0.67	8.08e−04
	RGS2	0.77	0.25	1	0.74	−0.65	2.42e−03
	MYD88	0.66	−0.28	1	0.63	0.48	3.16e−03
	IFITM2	0.81	−0.42	1	0.71	0.72	3.29e−03
	ID3	0.68	−0.37	9.56e−01	0.63	0.87	4.65e−03
	CD14	0.66	−0.49	1	0.63	1.39	5.37e−03
	SERPINA1	0.76	−0.65	1	0.63	1.27	6.64e−03

Top ten female-specific (top), male-specific (center) and candidate sex-dimorphic genes (bottom) obtained after the meta-analysis of *substantia nigra* Parkinson’s disease datasets. Functional annotations associated with these genes are found in Supplementary Table 9.

*Cst* Consistency score, i.e., the weighted proportion of the datasets supporting the cross-study log. fold change, *LFC* cross-study log. fold change, i.e., the weighted average base 2 log. fold change across the relevant datasets, *FDR* false-discovery rate, i.e., the *p* value adjusted for multiple testing.

^a^Gene with a potential confounder variable association (PMI/RIN), see details in Supplementary Note 2.

The meta-analysis used bulk transcriptomics data, and the relative proportions of cell types may therefore differ between the compared sample groups. Prior studies have shown that differential gene expression patterns derived from PD bulk transcriptomics datasets of cortical tissues can be confounded by cell type composition differences^42,43^. To estimate how this could affect our differential analysis, we analyzed the differential expression profiles of 38 selected cell type markers corresponding to 5 distinct brain cell types (more details about the markers can be found in Supplementary Note 1, Supplementary Table 13, and Supplementary Figs. 3–8). We observe that *TH*, a commonly used marker for dopaminergic neurons (DA), is the only cell-type marker significantly differentially expressed between patients and controls. This matches with prior expectations, as Parkinson’s disease is associated with a progressive loss of dopaminergic neurons. Since none of the other cell-type markers is differentially expressed, this suggests that the differential expression observed for other genes is unlikely to be driven significantly by differences in cell-type proportions (see also the complementary single-cell transcriptomics analyses below). In addition, we checked whether *substantia nigra* specific eQTLs from the GTEx database^44^ overlap with both the identified sex-associated DEGs and PD-associated GWAS variants, but this was not the case (see Supplementary Note 1).

Overall, many of the genes with significant sex-dependent PD-associated alterations are involved in cellular processes and organelles previously described to display pathological alterations in PD. In particular, they include genes involved in dopamine metabolism (*NR4A2*), lysosomal genes (*CXCR4*, *SGSH*), and mitochondrial genes (*NDUFA10*, *CA2*). Moreover, they cover genes previously implicated in PD relevant phenotypes such as dementia (*CXCR4*) and neurodegeneration (*MAPK1*) (see Discussion section for details on prior functional implications of these genes in PD and molecular sex differences).

### Pathway-level analysis of PD-associated molecular sex differences

To interpret sex-dependent molecular alterations in PD at the level of global shifts in cellular pathway and process activity, the significant DEGs derived from the statistical meta-analyses were further investigated using functional enrichment analyses across multiple pathway databases. For both the male and female analyses, we specifically analyzed pathway associations of the DEGs tagged as either sex-specific or sex-dimorphic. Selected enriched biological processes from the Gene Ontology (GO), Reactome, and Kyoto Encyclopedia of Genes and Genomes (KEGG) databases are presented in Table 2. Supplementary Tables 6 and 7 contain the complete pathway ranking results.

**Table 2:** Functional terms representative of the results of the functional enrichment analyses.

	Pathway identifier	Pathway name	Female gene ratio	Female FDR	Male gene ratio	Male FDR
Females	hsa04061	Viral protein interaction with cytokine and cytokine receptor	3/17	7.42e−02	1/283	9.93e−01
	hsa04062	Chemokine signaling pathway	3/17	1.71e−01	9/283	7.23e−01
	R-HSA-380108	Chemokine receptors bind chemokines	3/25	8.00e−02	–	–
	GO:0045236	CXCR chemokine receptor binding	2/35	1.62e−01	–	–
	GO:0006470	Protein dephosphorylation	5/34	2.25e−01	16/547	8.43e−01
Males	GO:0016469	Proton-transporting two-sector ATPase complex	–	–	8/557	4.82e−02
	hsa00020^a^	Citrate cycle (TCA cycle)	–	–	7/283	1.68e−02
	hsa04721	Synaptic vesicle cycle	–	–	12/283	4.30e−03
	hsa00190	Oxidative phosphorylation	1/17	3.60e−01	14/283	1.44e−02
	GO:0047496	Vesicle transport along microtubule	–	–	9/547	3.55e−02

For each gender, only the differentially expressed genes that are either gender-specific or gender-dimorphic are kept. The enrichment analysis was performed using a classical threshold-based approach. For each functional term, the results for both male and female analyses are displayed when available.

*Gene ratio* the number of DEGs annotated with the specific ontology term/the number of DEGs annotated with any term of that ontology, *FDR* false discovery rate, dashes indicate missing data.

^a^Pathway whose enrichment depends on genes with a potential confounder variable association (PMI/RIN), see details in Supplementary Note 2.

The pathway analysis results for the male-specific DEGs highlighted significantly enriched alterations in particular in mitochondria and energy metabolism related processes, such as *proton-transporting two-sector ATPase complex* (GO:0016469, FDR = 4.8e−2), *oxidative phosphorylation* (hsa00190, FDR = 1.4e−2) and *Citrate cycle* (hsa00020, FDR = 1.7e−2). These results are in line with prior observations of sex-specific differences in mitochondrial function (see refs. ^45–47^ and the “Discussion” section for the gene-level analyses) and the previous implication of mitochondrial impairment in PD^48,49^. Moreover, cellular processes related to synapses and associated signaling reactions were significantly enriched, including the *synaptic vesicle cycle* pathway in the KEGG database (hsa04721, FDR = 4.3e−3).

Given the lower number of significant female-specific DEGs, the analysis of the female-specific genes did not identify any significant functional enrichment after adjustment of the significance scores for multiple hypothesis testing. However, the top nominally significant functional gene sets are mostly associated with the inflammatory response, and more specifically with chemokine signaling, including processes such as *Chemokine signaling pathway* (hsa04061, FDR = 7.4e−2) and *Chemokine receptors bind chemokines* (R-HSA-380108, FDR = 8e−2). No significant overlap was observed between the pathways with female DEGs and male DEGs, suggesting that instead of affecting different genes in the same pathways, sex-specific changes tend to affect diverse pathways. Given a smaller number of female samples in the present meta-analysis, follow-up studies with larger numbers of female biospecimens are warranted to determine whether an enrichment of female-specific genes may be detectable in further cellular processes.

**Fig. 2:**
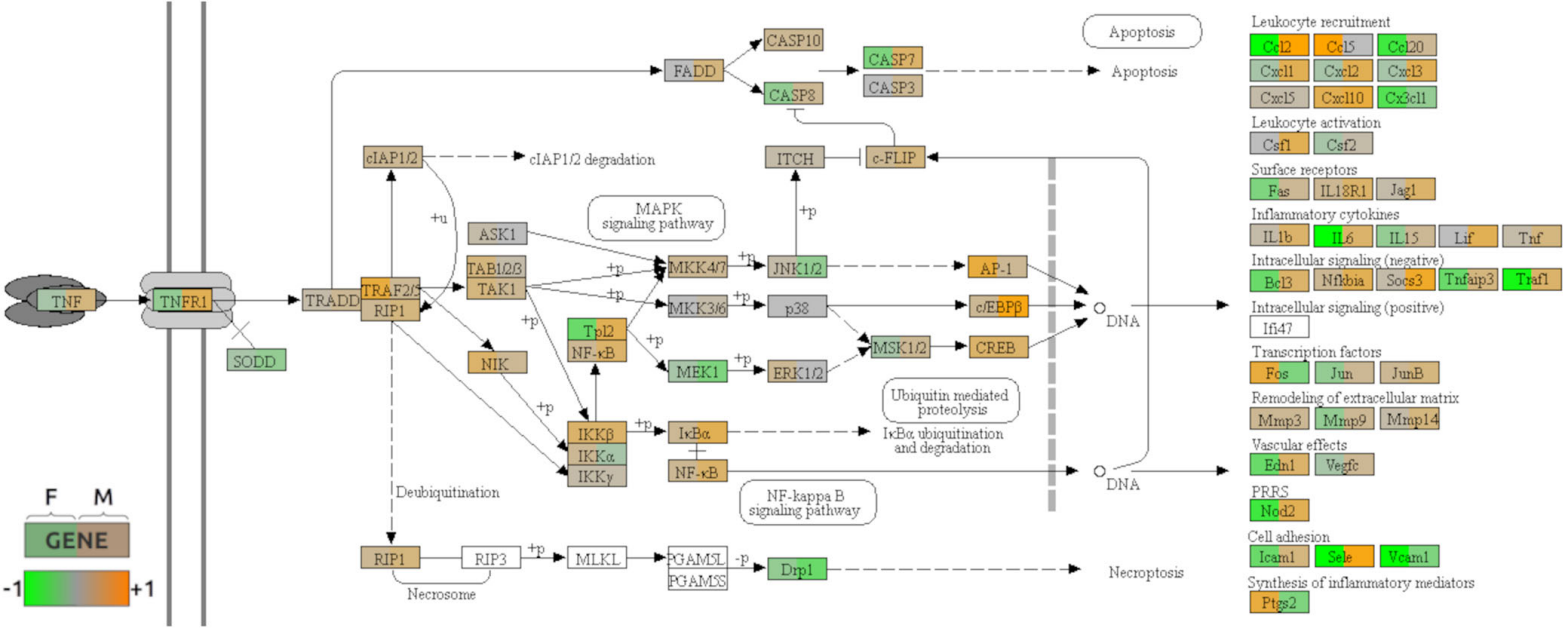
Overlay of the differential expression statistics on part of the map for the TNF signaling pathway. Each gene box has colors reflecting the cross-study log. fold change in the differential expression analyses for the female (left) and male analyses (right). Up- and down-expression in PD patients are respectively indicated by the orange and green color scales. The pathway map has been extracted from the KEGG database and the rendering was obtained using the software Pathview.

As a representative example illustration for coordinated sex-dependent expression alterations occurring in a cellular process, we show male and female differential expression profiles for the KEGG TNF signaling pathway in Fig. 2, where we observe that downstream targets are often associated with dimorphic differential expression patterns.

Overall, the pathway analyses revealed a significant enrichment of male-specific PD-associated DEGs in multiple processes previously implicated in PD, highlighting different top-ranked pathways for male and female DEGs. While male-specific DEGs were mainly over-represented in mitochondrial and energy metabolism related pathways, female-specific DEGs only showed nominally significant associations with inflammation and immune response related processes. Further detailed results from the functional enrichment analyses are described in Supplementary Note 1.

### Regulatory network analysis

**Fig. 3:**
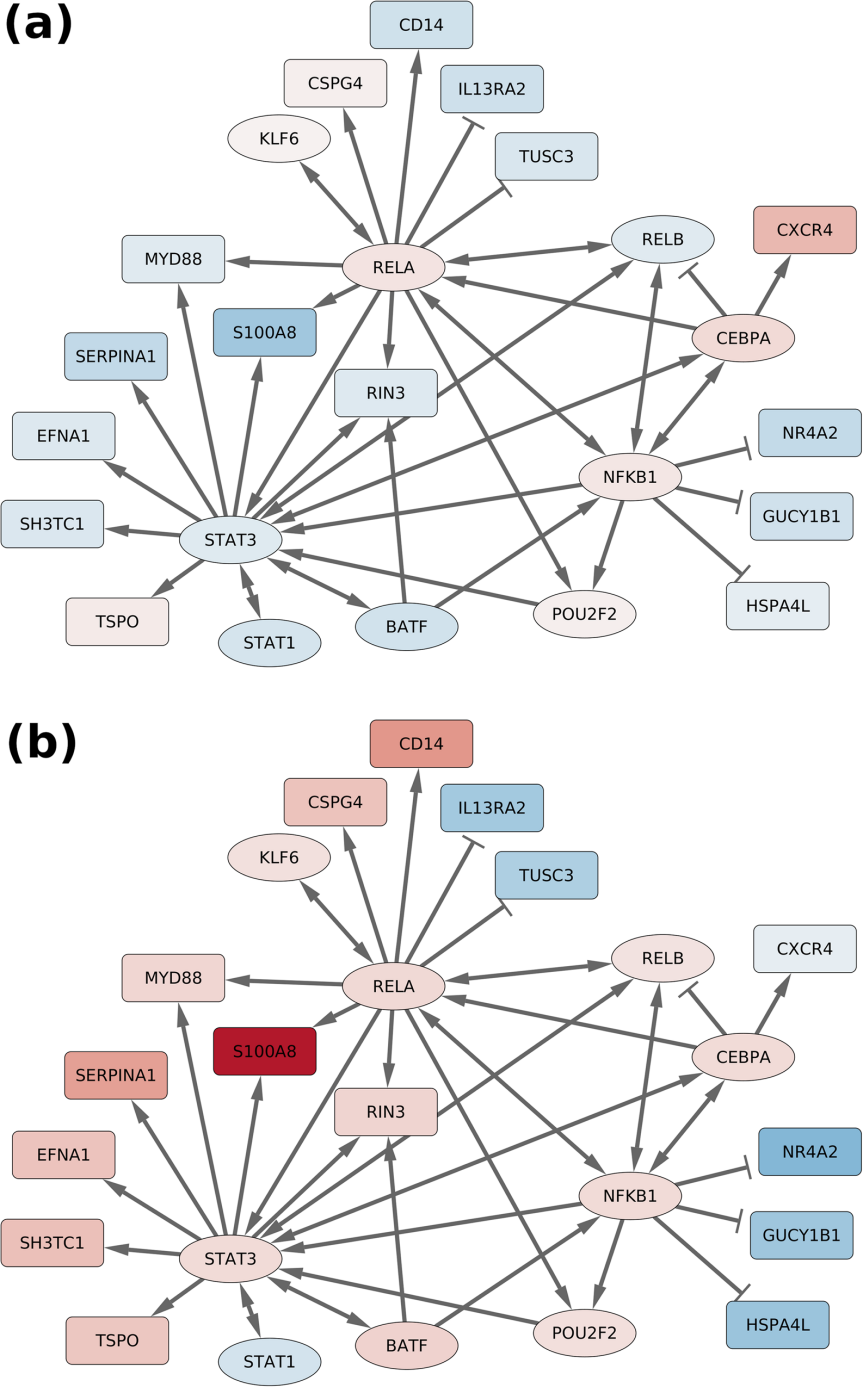
Visualization of the regulatory sub-networks centered around selected transcription factors. The transcription factors were selected based on the over-representation of their known target genes among the differentially expressed genes, favoring the factors whose predicted activity differs between females and males. Only the target genes with a minimum FDR (across the male and female analyses) below 0.01 are included. Both networks share the same content and layout, but the nodes are colored such that they reflect the cross-study log. fold change for the female (**a**) and male (**b**) analyses, respectively. The selected transcription factors are represented as ellipses, whereas the other genes are represented as boxes. A larger visualization of the network including more target genes (with a minimum FDR between 0.01 and 0.05) in the surrounding sub-network is presented in Supplementary Fig. 9).

In order to identify key transcription factors (TFs) that control many of the observed downstream sex-dependent expression changes in PD, the sex-specific DEGs were further investigated using target expression levels to estimate TF activity levels (see “Methods”). In total, we identified 18 TFs whose activity was estimated to differ between males and females. We observed that 13 of them displayed fold-changes across the male and female analyses which were consistent with the sex-dependent changes of their downstream targets. The top 10 selected TFs are presented in Table 3, the complete results are shown in Supplementary Table 8. We also reconstructed the regulatory network around these selected transcription factors to highlight specific regulatory mechanisms (see Fig. 3 and Supplementary Fig. 9). Interestingly, several of the transcription factors predicted to control sex-dependent gene regulatory mechanisms are members of the statin family (*STAT3*, *STAT1*) or members of the NF*κ*B complex (*NFKB1*, *REL*, *RELA* and *RELB*; see the discussion in section “Analysis of key transcription factors and molecular sub-networks”).

**Table 3:** Top ten transcription factors enriched in differentially expressed target genes.

Regulon	Transcription factor enrichment			Differential expression					
	Evidence level	Female predicted activity	Male predicted activity	Females			Males		
				Cst	LFC	FDR	Cst	LFC	FDR
NFKB1	A	−2.06	6.60	0.81	0.25	4.48e−01	0.87	0.42	6.18e−03
RELA	A	−1.16	6.01	0.88	0.29	9.00e−01	0.89	0.44	4.85e−01
STAT3	A	−1.30	5.73	0.85	−0.29	1	0.63	0.41	1.83e−01
STAT1	A	0.58	6.83	0.70	−0.42	6.93e−01	0.73	−0.43	4.09e−01
POU2F2	C	−2.02	4.19	0.62	0.13	1	0.59	0.36	1
CEBPA	A	−1.56	4.59	1.00	0.42	8.80e−01	0.52	0.41	8.43e−01
FOXO4	A	−2.49	2.62	0.55	0.51	6.82e−02	0.88	0.41	2.58e−01
KLF6	C	0.68	5.67	0.55	0.09	1	0.89	0.33	1.52e−01
RELB	C	−0.49	4.49	0.72	−0.28	1	0.89	0.30	9.08e−01
BATF	C	−1.60	3.36	0.91	−0.46	1	0.74	0.54	4.67e−01

The columns contain the results of the enrichment analysis (left) and the differential expression analysis (right). Functional annotations associated with these genes are found in Supplementary Table 9. The Omnipath/Dorothea evidence levels A, B, and C, respectively, represent high, likely, and medium confidences, see Figure 5A in ref. ^155^ for more details.

*Cst* Consistency score, *LFC* log. fold change, *FDR* false discovery rate.

### Cell-type-specific transcriptomic data analyses

The *post-mortem substantia nigra* samples considered in this study cover multiple different cell types, including dopaminergic neurons, astrocytes, oligodendrocytes and their progenitors, and microglial cells, among others^50–53^.

To investigate cell-type specific alterations, we therefore analyzed two single-cell transcriptomics datasets derived from *post-mortem substantia nigra* samples of PD patients and controls, performing dedicated differential expression and functional enrichment analyses for each cell type and on each dataset (see “Methods”). While the significantly smaller sample sizes compared to the bulk transcriptomic data analysis limited the number of detectable significant changes, the pathway analysis results highlighted sex-associated differential activities in mitochondrial processes, apoptosis and cytokine signaling, consistent with the results of the bulk analysis. These changes were particularly pronounced in oligodendrocytes and astrocytes, which may either suggest that these could be the main cell types affected by sex-dependent changes (see details in Supplementary Note 1 and Supplementary Tables 14−16) or reflect a greater power to detect variations in oligodendrocytes, since they represent 46%, and 51% respectively, of all cells in the two scRNA datasets. Moreover, we applied a deconvolution analysis to the bulk RNA-sequencing dataset (NBB), which confirmed the above-mentioned cell proportions. However, the same analysis is not feasible for the microarray datasets considered in this study (see details in Supplementary Note 1).

## Discussion

As a first general observation of the meta-analysis, many of the identified sex-specific and candidate sex-dimorphic genes are members of cellular processes previously implicated in PD pathogenesis and progression. In particular, the significant sex-dependent alterations affect dopamine metabolism (*NR4A2*), mitochondrial (*NDUFA10*, *CA2*) and lysosomal processes (*CXCR4*, *SGSH*), whose pathological alterations have been confirmed by several prior studies as common molecular hallmarks of PD^48,49,54–56^. However, the corresponding genes have previously not been associated with sex differences in the disease. Generic hormone-dependent sex differences have been reported for *NR4A2* expression, but they were only studied in white adipose tissue and not in the context of neurologic disorders^57^. Similarly, for *CXCR4*, estrogen was described to increase mRNA and protein levels, but this observation was limited to endometrial epithelial cells^58^.

No prior report of sex-specific differences were identified for the other top-ranked significant genes; however, an influence of biological sex on mitochondrial and lysosomal function in general is in line with the results from previous in vitro and in vivo studies across multiple cell types, covering data from healthy adult humans as well as various animal models^45–47,59–62^. Sex differences in the regulation of the autophagosome–lysosome system in particular have also been proposed to modulate the severity of neurodegenerative disorders, because prior studies suggest that women have a lower basal autophagy^61^.

Among the sex-associated DEGs with previously described regulatory functions in PD, the transcription factor *NR4A2* (Nuclear Receptor Subfamily 4 Group A Member 2; synonym: *NURR1*) stands out due to its key regulatory role in the maintenance of dopamine metabolism^63^ and inflammatory gene expression in glial cells^64^. As a member of the steroid-thyroid hormone-retinoid receptor superfamily, it controls the expression of many essential genes for the development of meso-diencephalic dopaminergic (mdDA) neurons, such as *SLC6A3*, *SLC18A2*, *TH* and *DRD2*. For all these genes, significant decreased expression values were observed, which are consistent with the upstream decrease in *NR4A2*. In addition, *NR4A2* down-regulation is significantly associated with adult human brain aging and has been demonstrated to increase the expression of the known PD-associated gene alpha-synuclein^65,66^. In the transcriptomics meta-analysis presented here, *NR4A2* generally displays lower expression in PD patients than in matched controls, but the expression change is significantly more pronounced in males than in females (females: LFC = −0.6, FDR = 1; males: LFC = −1.4, FDR = 1.6e−8).

Expression and abundance alterations of *NR4A2* have been associated with PD-like phenotypes and adult aging in both human and animal studies. Specifically, homozygous *NR4A2* knockout mice displayed Parkinsonism-like molecular phenotypes in PD-associated brain regions^67^, and heterozygous knockout mice were characterized by reduced locomotor activities^68^ and lower brain dopamine levels^69^.

Interestingly, a recent study showed that a synthetic ligand that activates *NR4A2* is neuroprotective in a mouse model of MPTP-Induced Parkinsonism, suppressing loss of dopaminergic neurons in the *substantia nigra pars compacta* and DA terminals in the striatum^64^. A further study found that activating compounds for *NR4A2* prevent neurotoxin (6-OHDA)-induced death in primary DA neurons and rat PC12 cells, and significantly ameliorate behavioral deficits (rotation behavior toward the lesion side) in a 6-OHDA lesioned rat model of PD without detectable dyskinesia-like side effects^70^. *NR4A2* heterodimerizes with Retinoid X receptor alpha (RXR*α*) in midbrain dopaminergic neurons, and synthetic ligands binding to the RXR*α* binding pocket were reported to confer neuroprotection in C57BL/6 mouse models using different toxins (6-OHDA, MPTP)^71^. Overall, these prior findings suggest that both genetic, environmental and sex-associated influences on *NR4A2* activity may be involved in modulating the risk and severity of PD. Given the prior evidence for the druggability of *NR4A2*, the beneficial effects of its activation in PD model systems, and the sex-dependent changes among its downstream target genes, the protein may warrant further investigation as target for pharmacological modulation of a subset of pathological molecular changes in PD that display sex-associated activity profiles.

A further gene of interest with PD-associated regulatory functions among the identified sex-dependent DEGs is *CA2* (Carbonic Anhydrase 2). It encodes an enzyme that catalyzes the reversible hydration of carbon dioxide, and is involved in mitochondrial pH regulation. Increased *CA2* levels in mitochondria have previously already been associated with neurodegeneration and aging. Specifically, an age-dependent increase of tissue-specific carbonic anhydrases in mitochondria has been observed in mouse brain samples, in particular in the Purkinje cell degeneration (pcd5J) mouse model^72^. The same study also showed that the exposure of *C. elegans* to *CA2* results in a dose-dependent shorter lifespan. Interestingly, many dopaminergic small molecule compounds (i.e., compounds with structure similar to the endogenous neurotransmitter dopamine, which are often used in the treatment of PD) are inhibitors of human carbonic anhydrases, such as *CA2*^73^, which may result in compensatory expression alterations in PD. Disambiguation between direct disease-associated effects and treatment effects on *CA2* expression will require further studies on drug-naïve patients. Regarding the observed sex differences, in the present meta-analysis, the over-expression of *CA2* in PD patients compared to matched controls is significantly stronger in females than in males (females: LFC = 1.1, FDR = 8.2e−4; males: LFC = 0.6, FDR = 0.5). No prior studies reporting sex differences in *CA2* levels in the brain could be identified; however, in prostate tissue from rats, a differential regulation of *CA2* by the hormones androgen and estrogen has been reported^74^.

Finally, the most significant candidate sex-dimorphic gene identified in the meta-analysis is *EFNA1* (Ephrin A1). This gene from the ephrin family binds to multiple ephrin-related receptors to mediate developmental events, in particular as part of nervous system development^75^. Interestingly, *EFNA1* was shown to mediate dopaminergic neurogenesis and angiogenesis in a rat model of PD, and its activation proposed as a potential target for the treatment of neurodegenerative diseases^76^. In the meta-analysis, *EFNA1* is significantly over-expressed in male PD patients compared to controls, whereas female patients display a slight, non-significant under-expression (females: LFC = −0.3, FDR = 1; males: LFC = 0.8, FDR = 9.3e−5). No previous reports on sex differences in *EFNA1* gene expression in the brain were identified.

In summary, the meta-analysis identified genes with statistically significant sex-specific or sex-dimorphic expression changes in PD samples compared to controls, including genes previously implicated in the regulation of dopamine metabolism, mitochondrial or lysosomal functions in the context of PD, and genes associated with general neurodegeneration or aging. An overview of the sex-dependent and PD-associated expression profiles for the three genes of interest discussed above, *NR4A2*, *CA2* and *EFNA1*, in representative transcriptomics datasets is presented in Fig. 1.

As a main result of the pathway analyses, we observed that DEGs which are either male-specific or sex-dimorphic are over-represented in mitochondrial and energy metabolism related pathways. Considering our results together with previous observations for other diseases involving mitochondrial dysfunction, such as Leber’s hereditary optic neuropathy (LHON), which also display a higher prevalence in males than in females as described for PD, this could indicate a potential generic increased vulnerability of males to mitochondrial impairments. This matches with findings from prior studies for healthy individuals showing a higher activity of mitochondrial respiratory complexes in females compared to males^77^. Furthermore, in this context, matrilineal inheritance of mitochondrial DNA (mtDNA) has previously been proposed to lead to a male-female asymmetry in the expected severity of mitochondrial diseases, because natural selection of mitochondria occurs only in females, and mitochondrial mutations are therefore expected to result more frequently in deleterious effects in men than women^78^. While damage in mtDNA has previously been proposed to be linked with PD and other diseases involving mitochondrial dysfunction^79,80^, the specific role of mtDNA in idiopathic PD is still unclear and conflicting results have been obtained from studies on mtDNA variation in PD^81^. Hormones, such as estrogen, also have important roles in the regulation of mitochondrial biogenesis and function^82^, and should therefore also be considered as potential contributing factor to sex-differences in mitochondrial function. Follow-up studies will be required to assess the specific influences of these different candidate factors on mitochondria-related sex differences in PD.

The most significant transcription factors identified in the network analysis are involved in inflammatory and immune response related processes (see Supplementary Table 9). A representative example is *STAT3* (Signal Transducer and Activator Of Transcription 3), which shows an non-significant increased expression in male patients (LFC = 0.41, FDR = 1.8e−1) and a non-significant decrease in female patients (LFC = −0.29, FDR = 1). Its target genes are enriched among the male DEGs (predicted activity = 5.73), and not among female DEGs (predicted activity = −1.3), in agreement with its known activating effect on its targets. *STAT3* is known to play a key regulatory role in determining the balance between astrogliogenesis and neurogenesis in brain neuroinflammation, and is activated in response to the pro-inflammatory cytokines IL-1*β* and TNF-*α*^83^. Multiple functional involvements of *STAT3* in PD-associated processes have previously been described, including the modulation of astrogliosis^84–87^, microglia activation^88–91^, and mitochondrial protein expression^92,93^. Interestingly, *STAT3* has been proposed as a potential drug target for neurodegenerative disorders, because its suppression during brain inflammation was found to promote neurogenesis and inhibit astrogliogenesis^83^.

Apart from cytokines, steroid hormones have also been described to influence *STAT3* response^94^, and correspondingly, *STAT3* activity has already been associated with sexual dimorphism in different organs, including the brain^92,95^. Moreover, *STAT3* activity correlates with clinical descriptors in a sex-dependent manner for different medical conditions, including brain cancer^96^ and inflammatory disorders of different organs^97–102^.

Among the other top-ranked TFs identified in the regulatory network analysis (see the top ten in Table 3) three of the five members of the NF-*κ*B family are also included: *NFKB1*, *RELA* and *RELB*. The targets of these three TFs are all enriched among the DEGs for males (predicted activity >4.4) and not for females (predicted activity <−0.4). Among the TFs themselves, one gene, *NFKB1*, also displays significantly increased expression in male patients (LFC = 0.42, FDR = 6.2e−3) and not in female patients (LFC = 0.25, FDR = 4.5e−1). The other two genes do not exhibit significant changes, which is in line with the common observation that TFs tend to display smaller alterations than their downstream targets, and the activity of these targets often provides a more robust indication of activated or deactivated regulatory mechanisms.

Due to its central role in the regulation of inflammation-associated processes, the NF-*κ*B pathway has previously also been investigated in the context of PD. Multiple sub-units of the NF-*κ*B complex were found to be over-expressed in the *substantia nigra* of PD patients^103,104^, where they are thought to enhance neuroinflammation^105,106^. In model organisms, NF-*κ*B activity correlates with the severity of PD-like symptoms and relevant cellular phenotypes, including mitochondrial homeostasis^107,108^. Therefore, *NFKB1* has also been proposed as a potential drug target for PD^104,109^. Regarding the potential mechanisms linking NF-*κ*B alterations to PD, previous studies have shown that *NFKB1* is regulated by the *PRKN* gene (Parkin), which harbors mutations associated with familial forms of PD^110^, and that *NFKB1* activity is also modulated by *STAT3*^111^ (see the discussion of *STAT3* above).

NF-*κ*B activity differs between males and females under a variety of physiological conditions in different organs^112–114^, including the brain^115^. In a cellular model, it was observed that *NFKB1* activation protects glutamatergic neurons against oxidative stress-induced neuronal death in a sex-dependant manner (with superior protection of neurons from female donors)^115^. In the context of brain disorders, genomic variants of the NF-*κ*B sub-unit *RELA* have been associated with schizophrenia in males^116^, but to the best our knowledge, NF-*κ*B family members have previously not been linked specifically to sexual dimorphism in PD.

Finally, we note that *NFKB1* inhibits the expression of *NR4A2*^117^, in line with NFKB1’s increased expression in male patients and the male-specific decrease in its downstream target genes. Similarly, the network in Fig. 3 also contains *EFNA1*, the top candidate sex-dimorphic gene, which is regulated by *STAT3*^118,119^.

This study has the following limitations: First, while the statistical power was sufficient to identify differentially expressed genes in both males and females after multiple testing correction, only a limited number of relevant samples were available to detect significant changes for smaller effect sizes, in particular for the female analysis. In total, our *substantia nigra* meta-analysis used 198 samples (after data processing and filtering). This is in particular impacting our definition of candidate sex-dimorphic genes, which is mainly based on effect size differences. Follow-up studies with greater statistical power and a more balanced representation to detect sexual dimorphism will have the potential to show statistical significance for a larger number of variations with small effect sizes. Moreover, the incomplete availability of metadata for most datasets limited our ability to filter out all potential effects of confounding factors. For instance, RNA integrity numbers (RIN) and post-mortem intervals (PMI) are available for only one, respectively two, of the datasets used for the main meta-analysis. Considering this constraint, we conducted dedicated confounder correlation analyses on these datasets (see Supplementary Note 1 and Supplementary Table 10), which suggest only limited influences of PMI/RIN on the presented results. Further follow-up analyses on larger-scale, fully annotated datasets (i.e., with complete metadata) will be required for additional independent validation of these results. Another limitation is linked to the technology used to measure the transcriptomics profiles (mostly microarrays and bulk RNA-sequencing). It has been demonstrated that cell type proportions can influence differential analyses in many different tissues, including brain tissues^42,43^. We conducted dedicated cell-type marker analyses to assess these potential influences in our meta-analysis, but cannot entirely rule out the possibility that cell type proportions differ more significantly between PD patients and healthy controls in the *substantia nigra* than these initial analyses suggest. Further research using single-cell technologies and large sample sizes will be required to better assess the potential role of differential cell-type proportions. A main strength of the applied methodology is that it integrates information from multiple different cohorts, experimental platforms and data types, and aggregates information from individual genes using pathway and network analyses. Thus, it provides both a transcriptome-wide ranking of gene-level sex-dependent alterations in PD and a global overview of the cellular processes impacted by these changes.

Follow-up studies will also be needed to more comprehensively characterize and confirm the mechanisms by which *NR4A2* and other regulatory genes control or modulate the identified molecular sex differences in PD. These could include targeted in vitro and in vivo perturbation experiments of *NR4A2* and other regulators with sex-dependent activity, such as *CA2* and *EFNA1*, to investigate their impact on PD molecular phenotypes in disease models for both males and females. If the druggability, sex-dependency, and neuroprotective roles of *NR4A2* or other candidate targets can be further substantiated, this could pave the way for subsequent preclinical investigations of adjuvant pharmacological strategies to reduce or alleviate sex-dependent molecular pathology in PD.

## Methods

**Fig. 4:**
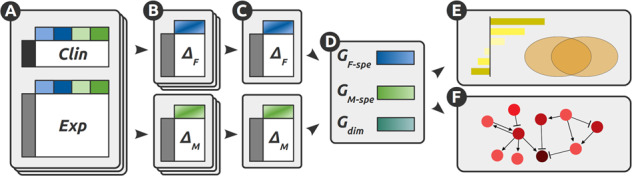
Global workflow of the analysis. **A** Relevant PD transcriptomics datasets were collected from public transcriptomic data repositories and complemented by an RNA-seq dataset generated in-house using samples from the Netherlands Brain Bank (see section “Data collection”). All datasets were curated and pre-processed (Clin: clinical data; Exp: experimental data; see “Methods”). **B** Two linear models were created to detect the genes differentially expressed between PD patients and controls, respectively for females (Δ_F_, top, blue) and males (Δ_M_, bottom, green). **C** For each model, a consensus was obtained through a meta-analysis across all datasets, favoring the genes with robust and reliable differential expression patterns. **D** Differentially expressed genes were then split into (i) female-specific genes (*G*_F−spe_, blue), (ii) male-specific genes (*G*_M−spe_, green) and (iii) candidate sex-dimorphic genes, whose directionality of the change differs between males and females (*G*_dim_, turquoise). **E** Functional enrichment of the selected differentially expressed genes to detect which biological processes are impacted. **F** Regulatory network analysis of the full set of genes, using experimentally validated interactions collected from multiple resources.

An overview of the entire processing and analysis workflow is presented in Fig. 4. Briefly, relevant Parkinson’s disease transcriptomics datasets were collected from public data repositories^120–136^ and complemented by a new dataset generated through RNA sequencing (RNA-seq) of samples from the Netherlands Brain Bank. All datasets were pre-processed and analyzed in order to detect significant transcript abundance differences between patients and controls (separately for males and females). Integrated differential expression statistics and rankings for each sex were obtained using a meta-analysis of all datasets. The genes with PD-associated expression alterations that differ significantly between males and females were further investigated using cellular pathway and network analyses. These analyses of bulk transcriptomic data were complemented by independent analyses of single-cell transcriptomics datasets, in order to further confirm the main identified pathway alterations and characterize their cell-type specificity. The following sections describe the methodologies for the data collection, processing, and downstream analyses.

### Sample preparation

*Post-mortem* human brain samples were obtained from The Netherlands Brain Bank, Netherlands Institute for Neuroscience (Amsterdam, The Netherlands). The tissue samples were homogenized (6875D Freezer/Mill Spex –Instrument Solutions Benelux BV). Fifty mg amount of sample was used for RNA isolation using the inhouse Tecan robot using AllPrep DNA/RNA/Protein kit (Qiagen) as mentioned before^137^. One μg of total RNA was used for library preparation using TruSeq stranded mRNA library preparation kit (Illumina) as per the protocol provided by the manufacturer. Briefly, the mRNA pull down was done using the magnetic beads with oligodT primer. To preserve the strand information, the second strand synthesis was done such that during PCR amplification only first strand was amplified. The libraries were quantified using Qubit dsDNA HS assay kit (Thermofisher) and the size distribution was determined using Agilent 2100 Bioanalyzer. Pooled libraries were sequenced at a the LCSB sequencing platform using NextSeq500. This dataset was integrated with pre-processed and quality-controlled transcriptomics data from public data repositories using a meta-analysis to increase the statistical power to detect expression changes and to ensure the robustness of the findings across data from different studies.

### Data collection

Relevant public transcriptomics datasets were identified through keyword-based searches in omics data repositories and literature databases. In particular, ArrayExpress (RRID: SCR_002964)^138^, Gene Expression Omnibus (GEO, RRID: SCR_005012)^139^, BioProject (RRID: SCR_004801)^140^ and relevant resources such as the Parkinson’s Progression Markers Initiative (PPMI)^141^, PubMed (RRID: SCR_004846) and Google Scholar (RRID: SCR_008878) were queried using the following keywords: “parkinson,” “substantia nigra,” and “dopaminergic” in combination with “transcriptomics,” “microarray,” and “RNA-seq.” The list of all online resources, together with the associated queries is provided in the Supplementary Table 1.

The retrieved datasets were further filtered by considering only human transcriptomics datasets (i) covering at least five thousand genes, (ii) focusing on both idiopathic PD patients and controls, and (iii) with at least ten samples. Only datasets analyzing the midbrain region *substantia nigra*, dopaminergic neurons from the *substantia nigra* or induced pluripotent stem cells derived dopaminergic neurons were retained. Two of the retrieved datasets (GSE42966 and GSE43490) were merged into a single dataset (Moreira) because they were generated by the same research group, from the same cohort, and shared most of their samples (13 and 15 samples each, 18 unique samples in total). Other types of brain tissues, such as the *cortex* or *putamen* were covered by an insufficient number of datasets to be further considered for a meta-analysis. The 20 selected transcriptomics datasets include 428 samples in total and were derived from 12 different measurement platforms^120–136^. Datasets focusing only on familial PD cases were not considered. Twelve, three and three datasets, respectively, covered the *substantia nigra* (SN), dopaminergic neurons from the *substantia nigra* (DA) and induced pluripotent stem cells derived dopaminergic neurons (iPSC-DA). The remaining two datasets are single-cell transcriptomics datasets (10× Genomics/Chromium) derived from *substantia nigra* samples from PD patients and controls (SC-SN), used to confirm key pathway alterations observed in the bulk transcriptomics data and to characterize their cell-type specificity. All datasets are described in further detail in Supplementary Tables 1–4.

We also ensured that there was no duplicate sample across the different datasets (i.e., samples derived from the same patient). By analyzing the dataset metadata, we identified two datasets with potential duplicates (GSE8397 and GSE26927), i.e., samples derived from the same brains stored at the UK Parkinson’s Disease Society Tissue Bank at Imperial College London. We therefore removed the five potential duplicated samples from the dataset GSE8397 prior to performing the meta-analysis.

For each platform, probe/transcript annotations, such as the chromosomal location and the corresponding gene names were retrieved from the Ensembl database (RRID: SCR_002344)^142^, whenever possible, and otherwise from the metadata provided with the dataset, and then matched using current Ensembl annotations for consistency (v102, Nov. 2020).

The original clinical annotations were extracted from the metadata obtained along with the datasets. Additional descriptors were provided by the data generators and missing values were predicted when possible (see next section).

### Missing value imputation

For some datasets, clinical descriptors, such as disease status, biological sex and age, were not available for all samples. Contacting the data generators allowed us to obtain 15 additional annotations for 14 samples. However, there were still missing values and we therefore investigated whether these could be predicted.

For eight samples across three datasets, the biological sex could not be retrieved. We therefore estimated these missing values by considering the expression levels of genes located on sex chromosomes. For each dataset, we selected loci that best discriminate between males and females on training data (i.e., samples associated with male or female patients), and used only these loci to make predictions for the samples for which no annotations were available. When using a leave-one-out cross-validation scheme, the predicted values matched with the already known annotations for two of the three datasets (accuracy was 100% and 85%, respectively, for GSE24378 and GSE20163), suggesting that, for these datasets, reliable predictions can be made for the few samples with missing annotations. This allowed us to update the clinical data with the predicted biological sex for five out of eight samples with missing information. However, for the remaining dataset with three sample annotations missing, the expression levels from sex chromosome genes did not provide sufficiently reliable estimations of the biological sex (accuracy for GSE20141 was 73%). Therefore, for this dataset, the three samples with unknown sex were simply removed. For some datasets, reliable predictions could be made, and potential annotation errors were detected (samples annotated as female but predicted to be male or vice versa) but these were not corrected.

Regarding age, the predictions derived from models based on the R package missRanger were not considered accurate enough to be considered. More precisely, and using a leave-one-out cross-validation scheme, the average RMSE (root mean square error) for the three datasets with missing ages was 11.3 years (values between 9.8 and 12.5). This means that the average difference between the real and predicted age was above 11 years, which was not considered acceptable given the important influence of age on expression data.

### Detecting experimental batches

The expression metadata was analyzed in order to detect the experimental batches. For the datasets extracted from the Gene Expression Omnibus (GEO) database (RRID: SCR_005012), the retrieved batches were harmonized with annotations from the Gemma database (RRID: SCR_008007)^143^.

For each dataset, we then checked whether there could be a potential batch artifact. Datasets with small batches (less than four samples) or with many batches (more than four) were not further investigated because it is difficult to take the batch effect into account without introducing some bias. For the remaining datasets, the experimental batches were included as covariate in the differential analysis. The summary of this analysis is presented in Supplementary Table 2.

### Age matching

The Parkinson’s disease cohorts usually contain age-matched patients and controls, however males and females might not always be age-matched as well, which is equally important in our study. We therefore investigated the age differences between (i) patients and controls, (ii) males and females and (iii) all four categories (female controls, female patients, male controls and male patients).

The results for all datasets are summarized in Supplementary Fig. 1. We observe a significant difference between the patient and control age values. This difference is less than five years for only four of the sixteen datasets (for two datasets, age is completely missing) and less than ten years for ten datasets. A similar situation is observed between males and females although there are some variations per dataset. The age distribution for the four categories of interest are plotted in Supplementary Fig. 1 for three representative datasets. The general trend is that patients are older than controls and males are younger than females. This motivated us to compare patients and controls for each sex independently instead of comparing the four categories of interest all at once with a more complex limma model (another motivation is that several datasets contain enough female samples but not enough male samples or vice versa). This also motivated us to include age as a covariate in the differential expression analysis when possible (see Supplementary Table 3 for more details about the linear models).

### Bulk data processing

For microarray datasets, a quality control analysis of the data was conducted using the R package ArrayQualityMetrics (v3.42.0, RRID: SCR_001335)^144^ prior to the raw data pre-processing in order to remove outlier samples. This package performs sample outlier detection analysis using three main methods. Any sample that was flagged as an outlier at least twice out of the three checks was removed from the analysis. In total, four samples from three datasets were removed according to these standard filtering criteria.

Affymetrix microarray datasets were then pre-processed using the Affymetrix PowerTools suite with the GC-RMA algorithm (v1.20.0)^145^. In general, the signal was summarized at the probe level, except for exon arrays, where a transcript level summary was computed. Illumina and Agilent microarray datasets were pre-processed using manufacturer-specific R software packages (beadarray v2.36.1 and limma v3.42.2)^146,147^.

The in-house generated RNA sequencing data was processed using the alignment-free quantification software Kallisto (v0.46)^148^ and the *homo sapiens* Ensembl v96 transcriptome, and then normalized by total read counts and mean-variance relationship estimation using voom^149^). For the RNA-sequencing data derived from GSE110717, only pre-processed data was available, and this dataset was therefore directly post-processed after quality control.

The post-processing always included the removal of samples from conditions other than idiopathic PD and healthy controls, of lowquality genetic probes, and of genetic probes/transcripts with zero variance. The heteroscedasticity of the measured signal intensities was plotted for all datasets and if variance-dependent signal intensities were observed, a variance stabilizing normalization was applied (R package vsn v3.54.0^150,151^).

The quality control analyses using ArrayQualityMetrics were repeated after completing these processing steps. Three additional samples were removed at this stage, according to the criteria described above, including two with sample quality metrics that were already close to the outlier thresholds in the initial quality control. After these preprocessing steps, the 18 filtered datasets contained in total 331 from the original 346 samples. Supplementary Table 1 contains a flow diagram that summarizes the number of datasets/samples that are kept after each preprocessing step, together with summary statistics on the demographics (e.g., disease status, sex, age).

### Removing irrelevant probes/transcripts

The lists of probes and transcripts were cleaned prior to the differential expression analyses in order to focus only on relevant entities. Probes and transcripts were selected using the same criteria but these are mostly relevant for probes as transcripts are in general well defined and associated with a single gene. First, probes associated with five genes or more were discarded as we wanted to focus on signals that are specific enough to be easily interpreted. Second, we also discarded the probes that match more than one gene if there also exists another probe that matches a subset of these genes (e.g., a probe that matches both gene A and gene B will be removed if there is another probe that matches only A or only B). Complex cases of probes matching overlapping sets of genes were removed as their interpretation would have been difficult (e.g., two probes matching respectively genes A+B and genes A+C will be removed regardless of the existence of probes matching only gene A or only gene B or only gene C).

### Differential expression analyses

Two differential expression analyses were performed on each dataset individually, using only female or male samples, to identify transcripts that display significant sex-dependent differences between Parkinson’s disease patients and controls.

For the processed bulk datasets, these differential analyses were conducted using the R package limma (v3.42.2, RRID: SCR_010943), which relies on linear models^147^. Available clinical covariates were included in the models in order to correct for potential confounders and biases and detect only the disease-associated biological alterations of interest. These covariates cover experimental batches, age and sample pairing information. Experimental sample batches were included as variable in the model when they confounded with the clinical outcomes and sufficient numbers of samples per batch enabled an estimation of possible batch effects (at least 4 samples per batch and maximum 4 batches in total, see section “Detecting experimental batches”). Information on the age of the subjects was also included in the model, whenever available, since ages were often not perfectly matched between patients and controls. The GSE8397 dataset contained two biological replicate samples per subject and the corresponding sample pairing information was also integrated into the model design. The configuration used for each linear model and for each dataset is described in more detail in the Supplementary Table 3.

### Dataset weights

The datasets were associated to weights in the meta-analysis. These weights represent a level of confidence and, as such, are derived from the number of samples for the current analysis based on the assumption that datasets with more samples are associated with larger, aka better detection powers and their differential expression analyses should therefore weight more in the meta-analysis. Only relevant samples are taken into account so that, for instance, the number of male samples is not considered for all female analyses. More precisely, the weights are derived from the lowest number of samples across the relevant patient categories (i.e., for a female analysis, this would mean the smallest between the number of female patients and the number of female controls). The weights are computed using an adaptation of the formula by Marot and Mayer (Eq. (1))^152^.

1 $$\begin{array}{ll}{\omega }_{d,s}\!\!&=\sqrt{\frac{{n}_{d,s}}{{\sum }_{\delta \in \Delta }{n}_{\delta ,s}}}\\ {{\mbox{with}}}\,{n}_{\delta ,s}&={\rm{min}}(| {\rm{patients}}_{\delta ,s}| ,| {\rm{controls}}_{\delta ,s}| )\end{array}$$

with *ω*_*d*,*s*_ the weight of dataset *d* for sex *s*, Δ the set of all omics datasets, patients_*δ*,*s*_ and controls_*δ*,*s*_ respectively the sets of patients and controls in dataset *δ* for sex *s*.

### Data integration

The statistical results for the differential expression analyses of the individual transcriptomics datasets were integrated using a meta-analysis across all pre-filtered datasets derived from the same tissue. For genes covered by multiple probes/transcripts, it was necessary to define the most relevant entity to select for the meta-analysis. We decided to select the probe/transcript with the highest average expression across the considered datasets since low-signal is associated with lower reliability. This means that, for a given gene, it is possible that two distinct probes/transcripts are selected for the male and female analyses. The list of the 211 genes for which this happens in at least one dataset can be found in Supplementary Table 5. These genes were not considered further in the present study. Prior to the meta-analysis, each gene is therefore associated with a base 2 log. fold change and a nominal *p* value per dataset.

Next, for each gene, the consensus activity shift (i.e., up- or down-expression) was determined through a weighted voting scheme, where the weights reflect the relative number of samples per dataset. Nominal *p* value significance scores obtained from the differential expression analyses on the individual datasets were then integrated using the weighted meta-analysis approach by Marot and Mayer^152^, again using weights corresponding to the relative number of samples per dataset. For each gene, the meta-analysis focused on the datasets with log. fold change consistent with the overall direction of the estimated cross-study log. fold change (see above) to ensure that the integrated *p* values represent gene activity shifts in the same direction. Finally, the resulting integrated meta-analysis *p* values were adjusted for multiple hypothesis testing using the Benjamini and Hochberg method^153^. The male and female analyses were run separately, adjusting however the nominal *p* values once using both sets of genes. A gene was considered significantly differentially expressed if the false-discovery rate was below 0.05.

We wanted to focus our meta-analysis on genes with a reliable signal. We therefore computed, for each gene, a reliability score by summing up the weights of the datasets for which the gene was present and dividing that value by the sum of all weights. If a given gene has no missing value, its reliability score is 1, if all values are missing, it is 0. In our case, genes whose reliability scores were below 2/3 were not considered further (i.e., representing a maximum of 33.34% missing values if all datasets were to have the same weights). This filter was implemented after the meta-analysis since it might not be fully *p* value independent. Additionally, apart from providing information on significance as main ranking criteria, we determined a cross-study estimate of the log. fold change for each gene by computing a weighted average of the log. fold changes that are consistent with the consensus activity shift. We also wanted to distinguish between genes that are consistently found to be differentially expressed (i.e., reported as up-expressed in 100% of the datasets) and genes with less clear patterns (i.e., reported as up-expressed in 51% of the datasets). A consistency score was established by computing the weighted percentage of datasets that report a log. fold change in the same direction than the cross-study log. fold change. Only genes with at least 60% consistency were taken into consideration for further analysis. In the evaluation of results, the reader should take into consideration both the final integrated significance score and the consistency score (see Tables 1 and 3 as well as Supplementary Tables 5, 11, and 12).

### Sex-specific and candidate sex-dimorphic genes

Next, the significantly differentially expressed genes (DEGs) with FDR < 0.05 in at least one analysis were categorized into female-specific, male-specific and candidate sex-dimorphic genes according to their differential expression profiles in males and in females. The overall process is described as a decision chart in Supplementary Fig. 2.

Due to the difference in detection power between the male and female analyses, the corresponding FDR values have different distributions (male FDR values are a lot smaller than female FDR values). This means that a categorization based only on FDR values will identify most male DEGs as male-specific and only few female DEGs as female-specific. We therefore decided to use a rank based strategy to define sex-specificity. More precisely, we have defined a sex-specificity index that is based on the rank ratios of the gene *π*-values^41^ (see Eq. (2)).

2 $$\begin{array}{l}{\rm{Spe}}_{g}\,=\,{\rm{rank}}\;{\rm{ratio}}({\pi }_{{\rm{F}},g})-{\rm{rank}}\;{\rm{ratio}}({\pi }_{{\rm{M}},g})\\ {{\mbox{with}}}\,{\pi }_{s,g}\,=\,-{\rm{log}}_{10}({\hat{p}}_{s,g})\times {\rm{abs}}({\rm{LFC}}_{s,g})\\ {{\mbox{with}}}\,{\hat{p}}_{s,g}\,=\,\left\{\begin{array}{ll}p-\epsilon \quad &\,{{\rm{if}}\;p = 1,}\,\\ p\quad &\,{{\mbox{otherwise.}}}\,\end{array}\right.\\ \end{array}$$

with *Spe*_*g*_ the sex-specificity score of gene *g*, *π*_*s*,*g*_ the *π* value of gene *g* for sex *s*, *LFC*_*s*,*g*_ and *p*_*s*,*g*_, respectively, the log. fold change and nominal *p* value of gene *g* obtained in the analysis for sex *s*. To avoid ties in the rank ratios, we introduced ${\hat{p}}_{s,g}$ (to avoid setting *π*_*s*,*g*_ to 0 when *p*_*s*,*g*_ is equal to 1) and relied on the nominal *p* value instead of the FDR.

Sex-specific DEGs were defined as genes that were only significantly differentially expressed in males (FDR < 0.05), but not approaching significance in females, or vice versa (based on their sex-specificity score *Spe*, see decision chart in Supplementary Fig. 2). The rationale was to avoid calling sex-specific a gene that is significant in males and close to significant in females (or vice versa), which would result in spurious assignments of sex-specificity. Candidate sex-dimorphic DEGs were defined as those with an opposite direction of the change between PD and control samples across males and females (up in one case and down in the other, as determined by the signs of the log. fold changes, and at least one of them showing statistically significant alteration) and requiring the genes to have a minimum absolute cross-study log. fold change >0.25 in both cases to ensure robustness (preventing misinterpretation of gene expression changes with small absolute effect sizes). Genes that showed consistent changes across the analyses, i.e., that were significantly differentially expressed for both males and females with the same direction of the change, were not further considered for subsequent sex-related analyses (for the interested reader, we provide a list of these shared genes in Supplementary Table 5).

### Cellular pathway and process analyses

Sex-dependent PD-associated cellular pathway and process alterations were determined using Fisher’s exact test to quantify the significance of the over-representation of sex-specific or sex-dimorphic DEGs among the members of each considered pathway. All genes associated with a reliable profile (i.e., not having more than 33.34% missing values) were used to build a relevant background. The resulting *p* value significance scores were then adjusted for multiple hypothesis testing according to the method by Benjamini and Hochberg. The pathway analysis was implemented using the *clusterProfiler* R package (v4.2.0, RRID: SCR_016884)^154^.

### Regulatory network analysis

Sex-dependent changes in gene regulatory sub-networks were identified in two steps. First, an enrichment analysis method was used to determine the transcription factors whose known target genes are over-represented among the DEGs. The enrichment is considered as a proxy of the transcription factor activities. It was computed using the strategy defined in *Dorothea*^155^ and implemented in the software *Funki*^156^, and the analysis was performed independently for male and female DEGs. The difference between the predicted activity for the sex-specific analyses was then used to identify the most relevant transcription factors involved in the control of downstream sex-dependent expression changes (i.e., transcription factors whose targets are enriched in one sex and relatively underrepresented in the other).

Regulatory interactions between the transcription factors of interest and the differentially expressed genes were extracted from two repositories, MetaCore GeneGo and OmniPath/Dorothea^155,157^ (OmniPath/Dorothea version of the 2022/09/01, GeneGo version of the 2022/09/01, RRID: SCR_008125). Gene names were unified through matching to official gene names from Ensembl (v107, Jul2022). The regulatory consistency of the interactions was further checked and interactions associated with inconsistent gene expression log. fold changes were filtered out (i.e., activated targets must show the same direction of change as the source gene, inhibited targets must show an opposite direction of change). Network visualizations presented in Fig. 3 and Supplementary Fig. 9 were created using Cytoscape^158^.

### Single-cell transcriptomics data analyses

Two single-cell RNA-seq datasets derived from *substantia nigra* samples of PD patients and controls were analyzed to confirm the main pathway analysis findings from the bulk transcriptomics analysis, and to study the cell-type specificity of sex-dependent disease-associated alterations. Both datasets were pre-processed and quality-filtered using the R software package Seurat (v3.2.0)^159^. Cells for which more than 10% of the counts mapped to ribosomal genes were removed after confirming that they also had lower numbers of total counts. In addition, outlier cells associated with less than 200 or more than 10,000 detected genes were also removed. The data was then normalized by applying the SC transform method^160^, using the mitochondrial gene/ribosomal gene/ribosomal RNA proportions, cell cycle estimates and patient age as covariates, whenever the relevant variables were available.

For datasets with existing metadata, the original cell cluster annotations corresponding to the different cell types were used, and otherwise, cell clusters were determined as follows. First, a PCA was run to extract the 50 most variable components. These were then used to create a cell-cell association network and identify cell clusters in this network using the enhanced Louvain algorithm (using multilevel refinement, with a resolution of 0.3, 500 different repetitions and maximum 500 iterations per repetition)^161^. Finally, the expression of known cell markers was used to estimate the most probable cell type for each cluster (see Supplementary Note 1). For each cell cluster, a differential expression analysis was performed separately for males and females using a Poisson distribution model^162^. Due to the limited number of available single-cell RNA-seq datasets, no integrative meta-analysis was performed, and the differentially expressed genes for each dataset were further investigated in separate functional enrichment analyses.

### Reporting summary

Further information on research design is available in the Nature Research Reporting Summary linked to this article.

## Supplementary information

Supplementary Material

Reporting Summary

## Data Availability

All data and materials associated with this publication, including links to the data as well as all the Supplementary Files mentioned in the manuscript, are hosted on a dedicated webpage (10.17881/hpbx-y095). For the RNA-seq data measured in-house, the *post-mortem* human brain samples were obtained from The Netherlands Brain Bank, Netherlands Institute for Neuroscience (Amsterdam, The Netherlands; open access: http://www.brainbank.nl). All material has been collected from donors for or from whom a written informed consent for a brain autopsy and the use of the material and clinical information for research purposes had been obtained by the NBB. The data can be accessed on the Gene Expression Omnibus database under the identifier GSE168496.

## Code availability

The analyses were implemented in R (v3.6.0) and bash (v4.4.20). The code of the entire analysis workflow is made available on the following GitLab repository under the MIT license: https://gitlab.lcsb.uni.lu/bds/geneder/geneder_core (tag corresponding to the version used in the manuscript: v1.3).
