# Supplementary material for: Systems level analysis of sex-dependent gene expression changes in Parkinson’s disease
Source: NPJ Parkinsons Dis. 2023 Jan 21;9:8. doi: 10.1038/s41531-023-00446-8 (PMC9867746; doi:10.1038/s41531-023-00446-8)
Supplement: Supplementary file 1 — Supplementary Material [file 41531_2023_446_MOESM1_ESM.pdf]

Supplementary Material to "*Systems level analysis of sex-dependent gene expression changes in Parkinson's disease*"

Léon-Charles Tranchevent, Rashi Halder, Enrico Glaab *

Supplementary Notes

1 Supplementary Note 1: Additional analyses 3

Supplementary Tables

1 Details about the collected datasets . . . 9
2 Experimental batch analysis summary . . . 10
3 Configuration of the differential expression models . . . 11
4 Ethnicity information associated with the *substantia nigra* datasets . . . 12
5 Meta-analysis results (SN) . . . 12
6 Functional enrichment (female analysis) . . . 13
7 Functional enrichment (male analysis) . . . 13
8 Transcription factor enrichment . . . 13
9 Function of highlighted genes . . . 13
10 Post-mortem interval and RNA integrity number analyses . . . 13
11 Meta-analysis results (DA) . . . 14
12 Meta-analysis results (iPSC-DA) . . . 14
13 Relevant biomarker genes . . . 14

*enrico.glaab@uni.lu, Luxembourg Centre for Systems Biomedicine (LCSB), University of Luxembourg, Esch-sur-Alzette, Luxembourg

14 Differential analysis (SC-SN: GSE157783) 14
15 Differential analysis (SC-SN: GSE178265) 15
16 Functional enrichment (SC-SN) 15

Supplementary Figures

1 Results of the age comparisons between patient groups 16
2 Sex-specific and sex-dimorphic gene identification procedure 17
3 Expression levels of GFAP (astrocytes) 18
4 Expression levels of TH (dopaminergic neurons) 19
5 Expression levels of GAD1 (GABAergic neurons) 20
6 Expression levels of ITGAM (microglia) 21
7 Expression levels of OLIG2 (oligodendrocytes) 22
8 Expression levels of cell type biomarkers 23
9 Enlarged visualization of the regulatory network 24

1 Supplementary Note 1: Additional analyses

Impact of subject ethnicity

Previous studies focusing on the investigation of clinical and symptomatic differences between male and female PD patients have reported a variety of significant sex disparities, *e.g.* associated with prevalence and incidence [1–6]. However, when considering distinct patient populations, the heterogeneity of different study cohorts has also led to diverse and partly conflicting observations. Overall, reported sex differences tend to be more pronounced in study cohorts from western populations compared to Asian populations. In particular, the incidence and disease trajectories for both sexes were more often reported to be similar in Asian populations [7–12], although some studies did not replicate these findings [13, 14].

We investigated the ethnicity of subjects included in our meta-analysis to assess the applicability of our results to different patient populations. Detailed information on subject ethnicity was only available for one dataset (GSE7621, which was ultimately not included in the *substantia nigra* analysis presented in the manuscript, because age annotations were missing). The original data generators could not provide further information on ethnicity. We therefore extracted information on the sources of the brain tissues from the relevant GEO records and associated publications to determine the geographic locations of the associated brain banks. The results showed that the majority of samples were either collected in the United States of America (USA, 114 samples, 47%) or northwestern Europe (United Kingdom, Netherlands, Germany and Belgium, 91 samples, 37%). The remaining samples are from Brazil (18 samples, 7%) or of unknown origin (21 samples, 9%; see detailed numbers in Table 4).

While the geographical information does not enable detailed and reliable conclusions on ethnicity distributions, the collected data suggest that Western ethnicities are strongly over-represented in the used data, while Asian and other ethnicities are underrepresented. Robust ethnicity-specific analyses for these underrepresented populations are therefore not feasible with the currently available data. Therefore, further meta-analyses for the currently underrepresented ethnicities will be required in the future in order to assess whether sex-specific and sex-dimorphic alterations in PD are shared across different populations.

Post-mortem interval and RNA integrity number analyses

Data for two potential confounding variables, the post-mortem interval (PMI) and the RNA integrity number (RIN) were not available for most datasets. We therefore could not include these variables directly as confounders in the limma models. However, for the datasets with available PMI values (*i.e.*, GSE26927 and NBB), we performed a dedicated Pearson correlation analysis, in order to identify specifically the genes whose expression correlates significantly with PMI. The correlation results of the two datasets were then integrated using the same meta-analysis procedure describe above. This analysis however did not reveal any significant gene (FDR < 0.05). We then investigated whether any of the top 100 non-significant genes whose nominal p-values was lower than 5% was also tagged as sex-specific or candidate sex-dimorphic by our main meta-analysis. Three of the 100 genes were identified as sex-specific or candidate sex-dimorphic (*AMD1*, *PPFIBP2*, and *C3orf18*). However, none of these genes is involved in pathways highlighted in the manuscript or in pathways associated with a significant adjusted p-value. Similarly, these genes have no influence on the regulatory network analyses (removing these three genes change neither the list of selected transcription factors nor the network visualizations).

We performed a similar analysis for the single dataset for which RIN scores were available for all samples (GSE26927). The correlation analysis revealed that the expression of 664 genes could be influenced by the RIN scores (Pearson correlation analysis; FDR < 0.05). We note that, for this particular dataset, the RIN scores correlate with the disease status. More precisely, controls are associated with lower RIN scores than PD patients, regardless of sex (point-biserial correlation; $r = 0.4$; p-value = 0.08), meaning that the influence of the RIN scores and the disease status cannot be distinguished clearly. However, among the 664 genes, only 6 genes are identified as male-specific (*i.e.*, *ACLY*, *BBS4*, *NCAM1*, *POU2F1*, *RRP1B* and *TAF1C*) and 1 as female-specific (*AMD1*, already highlighted in the PIM correlation analysis described above). There is no overlap with the list of candidate sex-dimorphic genes. We note that one gene, *ACLY*, is involved in the KEGG pathway 'TCA cycle'. However, similar to the PMI analysis, removing these genes does not change the qualitative results of the regulatory network analysis (both in terms of TF enrichment and in terms of network visualization results). The complete results of these correlation analyses are provided in Supplementary Table 10. The genes and pathways mentioned in the current section are marked with an asterisk in the main tables to highlight the potential confounder effect.

Sub-sampling male datasets

The *substantia nigra* datasets we have included in our meta-analysis are imbalanced (males are over-represented). To investigate the impact of this imbalance on our analysis, we ran three additional meta-analyses for which a proportion of male samples were removed as to have the same number of male and female samples per dataset. The sampling was performed randomly but otherwise the same pipeline was used (from quality control to meta-analysis). We then computed the Pearson correlation between the results of the three sub-sampling replicates and the results of the original meta-analysis. This was done using either the log. fold-changes or the negative decadic logarithm of the nominal p-values. In both cases, we observed relatively high correlation values (log. fold-changes: 0.69-0.73; p-values: 0.68-0.73). These results indicate that the effect of the male sample over-representation on our analysis is limited and is unlikely to explain the differential expression patterns we observe.

Overlap between DEGs and eQTLs

We investigated whether the differentially expressed genes identified in the current study overlap with known expression-QTLs and whether these were Parkinson's disease associated loci with confirmed genome-wide significance. First, 3,301 *substantia nigra* specific cis-eQTLs were collected from the GTEx database [15] (version of 2022/09/16). We then checked their overlap with the DEGs. We identified 12 variants that influence the expression of female DEGs and 123 for male DEGs (135 variants in total). When we restricted the analysis to the sex-specific and candidate sex-dimorphic genes, we retained 4, 65, and 3 variants that influence the expression of female-specific, male-specific, and candidate sex-dimorphic genes, respectively (for a total of 72 of the 135 variants). Next, we looked up whether any of these 135 variants had already been associated with PD, using *PDgene.org* [16] and recent meta-analyses of PD GWAS [17, 18]. Only GWAS variants with confirmed genome-wide significance were considered to avoid spurious findings. We identified one gene, *RAB29*, whose expression is influenced by rs3747973, a variant found in *PDgene.org*. This gene is differentially expressed in males, however it is neither male-specific nor sex-dimorphic. At the time of the analysis, GTEx did not contain any significant trans-eQTLs for the *substantia nigra*, so we could not perform a similar analysis for trans-eQTLs. Finally, we also determined the overlap between the sex-associated DEGs and the variants from *PDgene.org* and the GWAS meta-analyses (excluding the eQTLs from the comparison). We identified eight male-specific genes that harbor PD relevant variants (*SNCA*, *ABCB9*, *ITPKB*, *NSF*, *R3HDM1*, *RIMS1*, *SCAMP5*,

SH3GL2), including the PD-mutated gene *SNCA*, which encodes α-synuclein, the main protein component of Parkinson's hallmark Lewy bodies. There was however no overlap for the smaller number of female-specific and candidate sex-dimorphic genes.

Sex-specific differences in glycosylation

Recently, sex-specific differences in glycosylation have been reported in the plasma of PD patients [19]. The authors measured the levels of advanced glycation end products, such as carboxymethyl-lysine, that are known biomarkers of several age-related disorders including neuro-degenerative diseases [20].

We wondered whether such differences could also be observed at the transcriptional level in the *substantia nigra*. Our analysis relies on a set of 80 genes designed specifically to evaluate glycosylation activity at the transcriptional level [21]. The male DEGs, but not the female DEGs (only 1 of the 80 genes is differentially expressed), are weakly enriched in glycosylation genes (Fisher's exact test; females: p-value = N/A; males: p-value = 5.0e-2). This includes *B4GALT1*, a gene that is up-expressed in males (LFC = 0.37, FDR = 4.1e-2) but not in females (LFC = -0.19, FDR = 1). When we considered the relevant Gene Ontology term (GO:0006486, 868 genes), we did not detect any functional enrichment neither in males nor in females. We hypothesize that it could be because the Gene Ontology term is based on a less restrictive definition and is therefore associated with a lot of genes that are not really key glycosylation players.

Analysis of cell-type specific datasets

The *post-mortem substantia nigra* samples contain many different cell types and therefore the differential expression profiles we observe may be caused by different cell types in the tissue, including but not restricted to astrocytes, oligodendrocytes and their progenitors and microglial cells [22–25]. We therefore set to investigate the profiles of the same genes in *post-mortem* dopaminergic neurons (DA) and induced pluripotent stem cells derived dopaminergic neurons (iPSC-DA). The results of the meta-analyses corresponding to these two tissues can be found in Supplementary Tables 11 and 12.

Briefly, there are far less samples (both male and female) for these two analyses when compared to the main analyses based on the *substantia nigra*. As a consequence, there are no differentially expressed gene in DA / iPSC-DA. In addition, we report that none of the genes that were significantly differentially expressed in SN are top ranked in either DA or iPSC-DA. This could indicate

that the variations we observe in SN are indeed coming from non neuronal cells such as astrocytes, oligodendrocytes and microglia. Alternatively, it could also be due to the very low samples sizes, and further studies will be necessary to better understand the mechanisms at play and whether oxidative stress and cell death are also perturbed in neurons as reported previously [26, 27].

Potential biomarkers corresponding to the most prevalent cell types found in the *substantia nigra* were identified from relevant online resources [1,2], literature reviews and scientific publications [28–33]. The expression profiles of these potential biomarkers were checked against large-scale cell type specific transcriptomics datasets to insure that their expression was indeed cell type specific [3,4]. In total, 38 potential biomarkers were retained for 5 cell types including dopaminergic neurons, GABAergic neurons, oligodendrocytes (including progenitors), astrocytes and microglia (see Table 13). Their expression profiles were checked for all datasets and for all sample categories of interest (see examples in Figures 3-7). *TH* is the only gene that is found to be differentially expressed between patients and controls. The other biomarkers have similar profiles between males and females suggesting that the evolution of cell type proportions are similar between both sexes.

The expression levels of relevant biomarkers across the SN, DA and iPSC-DA collections are shown in Supplementary Figure 8. The biomarker profiles are not always perfectly clear (*i.e.*, non neuronal biomarkers are still expressed in DA samples) because most of the data is extracted from microarray datasets that are known to be susceptible to noise. However, these indicate that the SN samples indeed contain different cell types besides neurons and that neurons themselves might not represent the most abundant cell type, which is in line with recent single-cell analyses of SN samples in human or animal models [22–25]. It has been reported that PD relevant transcriptomics variations are not restricted to a single brain cell type [23] and that bulk transcriptome measurements might not represent the most suitable tool to clearly associate the observed variations to distinct cell types [34]. Therefore, additional experiments on these cell types, including via single cell analysis or through advanced modeling techniques [34], are necessary to better understand the molecular mechanisms underlying the sex-specific and sex-dimorphic variations.

We also investigated two single-cell transcriptomics datasets of the *substantia nigra* tissue covering respectively 41k and 255k cells from 11 and 71 PD patients and controls. Since there are only two datasets, we could not perform a proper meta-analysis and instead relied on a simple differential expression analysis (again separately for males and females) followed by a functional

[1] https://www.abcam.com/neuroscience/dopaminergic-neurons-and-their-major-markers
[2] https://docs.abcam.com/pdf/neuroscience/neural-lineage-markers-web.pdf
[3] http://www.brainrnaseq.org/
[4] http://genetics.wustl.edu/jdlab/csea-tool-2/

enrichment analysis of the sex-specific and candidate sex-dimorphic genes (using Fisher's exact test). These analyses are run separately per cell type in order to detect the variations associated with neuronal and non neuronal cells.

At the gene level, we observe significant variations between patients and controls for all major brain cell types, both sexes and both datasets (see Supplementary Table 14 and 15). There seems to be however little to no overlap between the differentially expressed genes of the SN meta-analysis and the ones observed in the single-cell SN datasets. At the pathway level however, the results indicate that similar pathways are perturbed (see Supplementary Table 16). For instance, male-specific oligodendrocyte and sex-dimorphic astrocyte genes are enriched in mitochondrial related processes, cell death, neuro-inflammation (in particular cytokine signaling) and even NFκB signaling. We note that, in line with prior expectations, the variations shared between the SN meta-analysis and the scRNA analysis are observed in the most prevalent cell types: oligodendrocytes (which represent respectively 46% and 51% of all cells) and astrocytes (which represent 8-11% of all cells, still within the range of other cell types such as OPCs and microglia, 7-9%). The results of the female-specific are a less clear which, once again, could be due to the low number of female cells / samples. This only reinforces the necessity to conduct additional analyses on sex-matched cohorts.

Deconvolution analyses

A deconvolution analysis was applied to the bulk RNA-sequencing dataset (NBB) using CibersortX [35]. We used the online interface of Cibertsortx to estimate the cell-type proportions, using a signature derived from the single-cell dataset GSE157783 (for 12 cell types). The signature genes were selected through a global differential expression analysis (comparing each cell type versus all others) and by keeping only the genes associated with a FDR < 0.01, an absolute cross-study log. fold-change > 1, and a maximum count of 50; limiting the number of genes selected per cell type to 75 (for a total of max 900 genes for the 12 cell types covered). The final signature consisted of 784 genes. Using the online version of Cibertsortx, we selected the custom mode of the 'Impute cell fractions analysis' module with the following parameters: quantile normalization disabled, no absolute mode, 100 permutations. We tried both not using any batch normalization method or using the B mode and report the values obtained for both runs. The results are compatible with the proportions of single-cell datasets and indicate that oligodendrocytes are the most prevalent cells, with an estimated proportion of 45-47%, while dopaminergic neurons represent between 2% and 3% of all cells. We could not run a deconvolution analysis on the microarray datasets, since relevant

single cell data for microarrays (required to create the necessary signature) was not available.

Table is too large to be displayed.
It is hosted on a dedicated webpage (https://doi.org/10.17881/hpbx-y095)

Supplementary Table 1: Details about the collected datasets. This table lists the online resources we have queried to collect transcriptomic datasets relevant for our study. Each resource is associated with a URL, a query and its filters. There is no query/filter for the brain banks as their publication list was manually mined. It also contains the list of datasets collected for the meta-analyses. The datasets are categorized according to the investigated tissues (*substantia nigra*, dopaminergic neurons and induced pluripotent stem cells derived dopaminergic neurons). The table also contains the lists of samples that were discarded or for which the metadata was modified during the analysis.

Dataset	Exp. batches	Confounder (heatmap)	Confounder (PCA factors)	Covariate
GSE20141	5,6,7	Yes	Batch	Yes
GSE20163	2,2,13	Yes	Disease status	No
GSE20164	4,7	Yes	Batch	Yes
GSE20292	1,2,2,2,4,5,13	Yes	No	No
GSE20333	2,4,6	No	-	No
GSE7307	7,15	Yes	Tissue	Yes
GSE7621	1,1,1,1,1,1,2,2,2,3,3,3,4	No	-	No
GSE8397	2,4,4,4,7,8,10	Yes	No	No
Simunovic	6,13	Yes	Batch	Yes
GSE24378	6,11	Yes	Batch	Yes
Moreira	-	Yes	No	No
GSE26927	-	Yes	No	No
GSE20159	9,12,12	Yes	Batch	Yes
GSE49036	1,3,4,4,5	Yes	Disease status	No
NBB	16	-	-	No
GSE110717	15	-	-	No
GSE51922	10	-	-	No
GSE99253	8	-	-	No
GSE157783	11	-	-	No
GSE178265	71	-	-	No

Supplementary Table 2: Experimental batch analysis summary. For each dataset, the retrieved batches are indicated, as well as whether the batches correlate with patterns in the expression heatmaps or with factors obtained via PCA. The last column indicates whether batches are included as covariate in the limma models.

Dataset	Tissue	Sex	VSN	Age	Batch	Paired tissue(1)
GSE20159	SN	F,M	✔	✔	✔	
GSE20163	SN	F,M	✔			
GSE20164	SN	F	✔	✔	✔	
GSE20292	SN	F,M	✔	✔		
GSE20333	SN	(2)	✔	✔		
GSE26927	SN	F,M	✔	✔		
GSE49036	SN	F,M	✔	✔		
GSE7307	SN	M	✔		✔	
GSE7621	SN	F,M	✔			
GSE8397	SN	F,M	✔	✔		✔
Moreira	SN	M	✔	✔		
NBB	SN	F,M		✔		
GSE20141	DA	F,M	✔	✔	✔	
GSE24378	DA	F,M	✔		✔	
Simunovic	DA	F,M	✔	✔	✔	
GSE110717	iPSC-DA	M		✔		
GSE51922	iPSC-DA	F,M	✔	✔		
GSE99253	iPSC-DA	M	✔	✔		
GSE157783	SC-SN	F,M				
GSE178265	SC-SN	F,M		✔		

Supplementary Table 3: Configuration of the differential expression models. Each dataset is associated with a model that relies on specific covariates such as age and experimental batch. In addition, a model was used either for the female analysis (F), the male analysis (M) or both (F,M). The VSN column indicates whether variance normalization was performed to control the heteroscedasticity. (1) The 'paired tissue' covariate was created for a dataset that contains paired samples, *i.e.*, for which two distinct samples from two different *substantia nigra* regions were extracted per brain. (2) After processing, the GSE20333 dataset was discarded because it did not contain enough male controls nor enough female patients. [SN: *substantia nigra*, DA: dopaminergic neurons, iPSC-DA: induced pluripotent stem cells derived dopaminergic neurons, SC-SN: single-cell and *substantia nigra*]

Dataset	No. samples	Ethnicity	Brain tissue origin
GSE20163	17	-	USA
GSE20164	10	-	USA
GSE20292	29	-	USA
GSE7307	21	-	-
GSE7621	25	White	USA
GSE8397	38	-	United Kingdom (-), Belgium (-)
Moreira	18	-	Brazil
GSE26927	20	-	United Kingdom (14), Germany (6)
GSE20159	33	-	USA
GSE49036	17	-	The Netherlands
NBB	16	-	The Netherlands

Supplementary Table 4: Ethnicity information associated with the *substantia nigra* datasets. For each dataset, the number of samples (after quality control and filtering), the ethnicity values (as provided in the clinical data) and the country in which the brain tissues were collected are indicated. When multiple countries are listed, the numbers in the parentheses indicate the relevant numbers of samples per country, if available. Dashes indicate missing data.

Table is too large to be displayed.
It is hosted on a dedicated webpage (https://doi.org/10.17881/hpbx-y095)

Supplementary Table 5: Meta-analysis results (SN). This table contains the complete results for the main meta-analysis (*i.e.*, for the *substantia nigra* samples), that is the integration of the differential expression analyses across all relevant datasets. The results are presented in six different spreadsheets corresponding to the two differential analyses (female and male) and the four categories of genes (female- and male-specific, sex-dimorphic and sex-neutral). It also contains the names of the genes that were matched to different probesets / transcripts in the male and female analyses and whose differential expression should therefore not be directly compared between the two analyses.

Table is too large to be displayed.

It is hosted on a dedicated webpage (https://doi.org/10.17881/hpbx-y095)

Supplementary Table 6: Functional enrichment (female analysis). Complete results of the functional enrichment analysis for the female-specific genes using Fisher's exact test. The results are presented in five spreadsheets corresponding to five different ontologies (GO-BP, GO-CC, GO-MF, KEGG and REACTOME).

Table is too large to be displayed.

It is hosted on a dedicated webpage (https://doi.org/10.17881/hpbx-y095)

Supplementary Table 7: Functional enrichment (male analysis). Complete results of the functional enrichment analysis for the male-specific genes using Fisher's exact test. The results are presented in five spreadsheets corresponding to five different ontologies (GO-BP, GO-CC, GO-MF, KEGG and REACTOME).

Table is too large to be displayed.

It is hosted on a dedicated webpage (https://doi.org/10.17881/hpbx-y095)

Supplementary Table 8: Transcription factor enrichment. Complete results for the transcription factor enrichment analysis. The results are presented in a single spreadsheet that combines both the male and female analyses.

Table is too large to be displayed.

It is hosted on a dedicated webpage (https://doi.org/10.17881/hpbx-y095)

Supplementary Table 9: Function of highlighted genes. It contains the functional annotations of the genes presented in main Tables 1 and 3

Table is too large to be displayed.

It is hosted on a dedicated webpage (https://doi.org/10.17881/hpbx-y095)

Supplementary Table 10: Post-mortem interval and RNA integrity number analyses. It contains the results of the correlation analyses between expression profiles and PMI or RIN values.

Table is too large to be displayed.

It is hosted on a dedicated webpage (https://doi.org/10.17881/hpbx-y095)

Supplementary Table 11: Meta-analysis results (DA). Results for the meta-analysis of the dopaminergic neuron samples (DA), that is the integration of the differential expression analyses across all relevant datasets. The results are presented in two different spreadsheets corresponding to the two differential analyses (female and male).

Table is too large to be displayed.

It is hosted on a dedicated webpage (https://doi.org/10.17881/hpbx-y095)

Supplementary Table 12: Meta-analysis results (iPSC-DA). Results for the meta-analysis of the induced pluripotent stem cell derived dopaminergic neuron samples (iPSC-DA), that is the integration of the differential expression analyses across all relevant datasets. The results are presented in two different spreadsheets corresponding to the two differential analyses (female and male).

Cell type	Gene names
Astrocytes	ALDH1L1, AQP4, EAAT1, EAAT2, FGFR3, GFAP, GJB6, GLAST, GLT-1, GLUL, SLC1A2, SLC1A3
Dopaminergic neurons	DAT, FOXA2, LMX1B, SLC6A3, TH
GABAergic neurons	GAD1, GAD2, GAD65, GAD67
Microglia	CD11B, CD45, CD68, ITGAM, PTPRC, TMEM119
Oligodendrocytes (incl. progenitors)	CLDN11, GJC2, MBP, MOG, OLIG1, OLIG2, OSP, PDGFRA, PLP1, SOX10, UGT8

Supplementary Table 13: Relevant biomarker genes. These genes are used as biomarkers in the present study to characterize the proportion of brain cell types in selected transcriptomics datasets.

Table is too large to be displayed.

It is hosted on a dedicated webpage (https://doi.org/10.17881/hpbx-y095)

Supplementary Table 14: Differential analysis (SC-SN: GSE157783). Results for the differential analysis of the single-cell *substantia nigra* dataset GSE157783 (SC-SN). The results are presented in twelve different spreadsheets corresponding to the distinct differential analyses (three per cell type, four cell types included).

Table is too large to be displayed.
It is hosted on a dedicated webpage (https://doi.org/10.17881/hpbx-y095)

Supplementary Table 15: Differential analysis (SC-SN: GSE178265). Results for the differential analysis of the single-cell *substantia nigra* dataset GSE178265 (SC-SN). The results are presented in twenty four different spreadsheets corresponding to the distinct differential analyses (three per cell type, eight cell types included).

Table is too large to be displayed.
It is hosted on a dedicated webpage (https://doi.org/10.17881/hpbx-y095)

Supplementary Table 16: Functional enrichment (SC-SN). This archive contains the complete results of the functional enrichment analysis for the sex-specific and candidate sex-dimorphic genes for the SC-SN datasets (GSE157783 and GSE178265) using Fisher's exact test. Each file contains the results for a given configuration (*i.e.*, dataset / cell type / gene set / ontology)

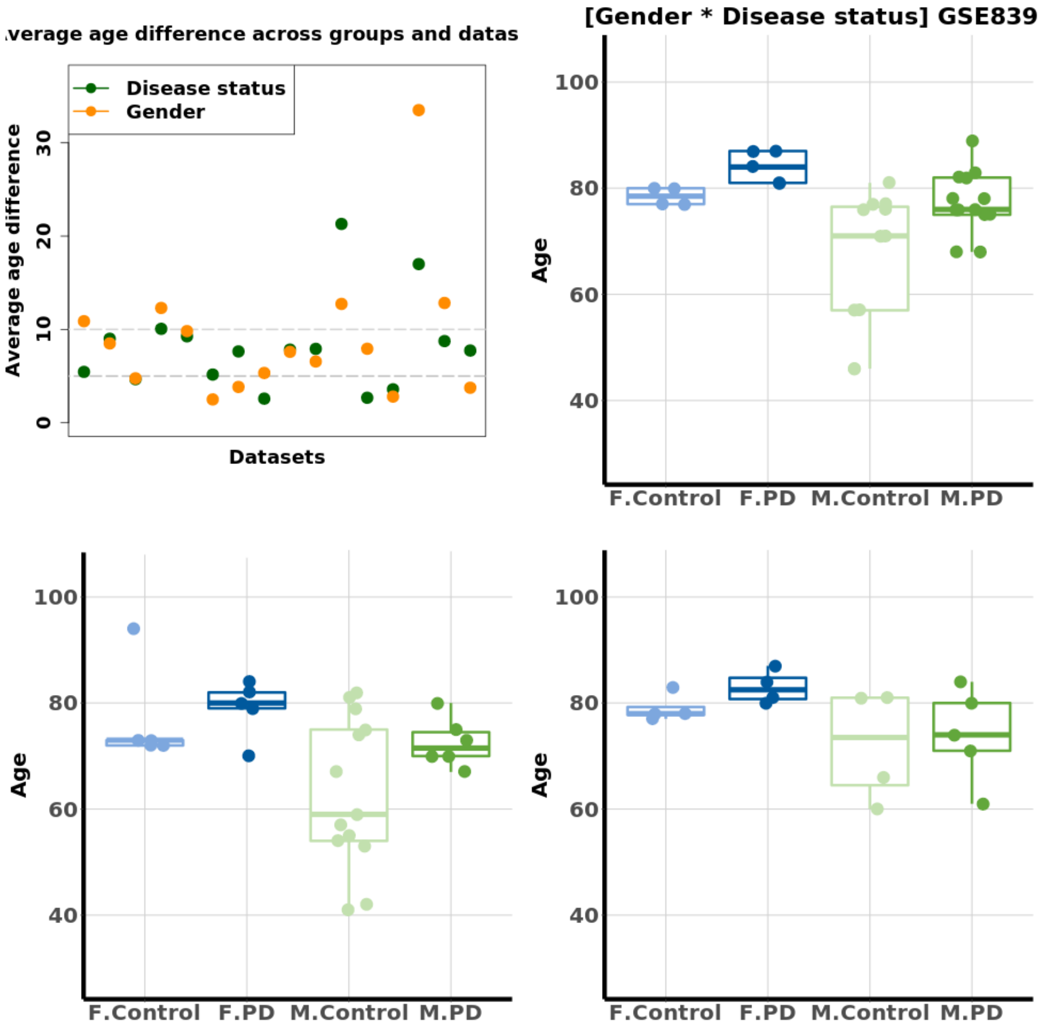

Supplementary Figure 1: Results of the age comparisons between patient groups. (Top left) Global summary of the analysis. The average age difference (x-axis) is plotted for all datasets (y-axis) comparing patients and controls (green dots) and males and females (orange dots). Two dotted lines indicate respectively five and ten years. (Top right and bottom) Age distributions as boxplots between the four categories of interest (female controls, female patients, male controls and male patients; x-axis) and for three representative datasets, respectively GSE8397 (top right), GSE20292 (bottom left) and GSE49036 (bottom right). Each boxplot is represented by the median, two hinges (representing the first and third quartiles) and two whiskers (extending up to 1.5 * inter-quartile range from the hinges).

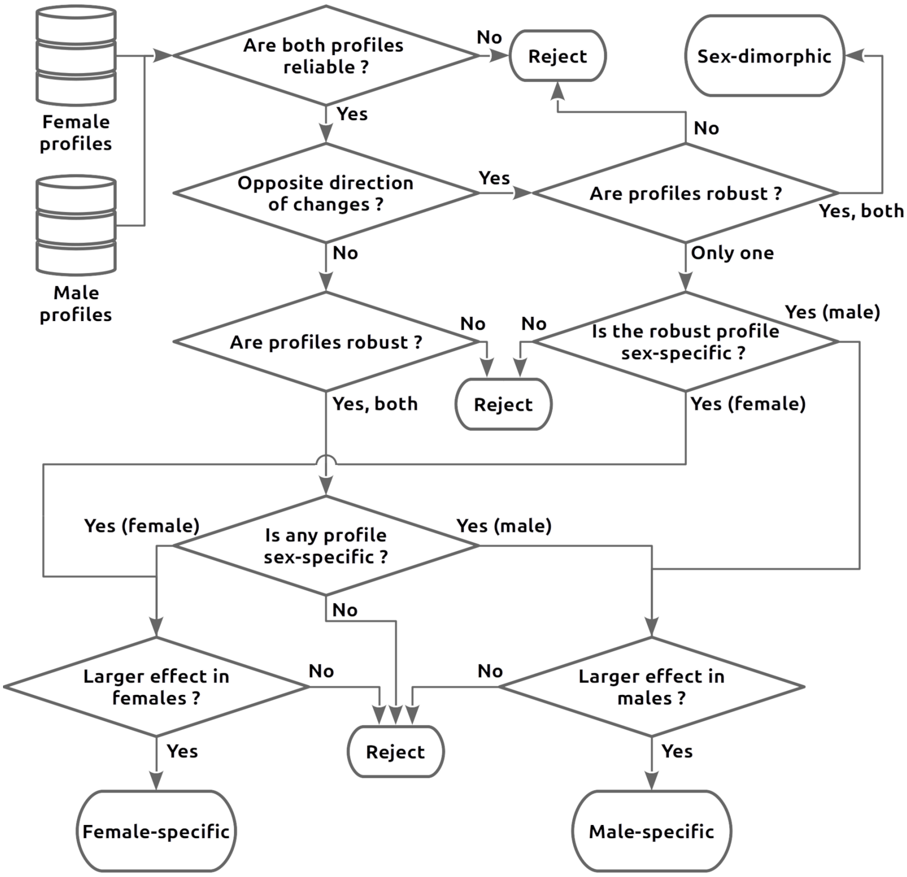

Supplementary Figure 2: Sex-specific and sex-dimorphic gene identification procedure. For each gene, male and female profiles are extracted from the meta-analysis. Each profile consists of a cross-study log. fold-change, a p-value, a FDR value and two tags representing the reliability and consistency of the profile. These values are used in various tests (depicted) to identify the genes that are potentially sex-specific or potentially sex-dimorphic. 'Opposite direction of changes' refers to whether the LFC from the male and female analyses are of different sign while being both greater than 0.25 in absolute value, suggesting potential dimorphism. 'Is any profile sex-specific' refers to whether the sex-specificity index (Spe_g) is greater than 10% for either males or females. 'Larger effect in females' refers to whether the LFC in females is actually larger than the LFC in males (in absolute value).

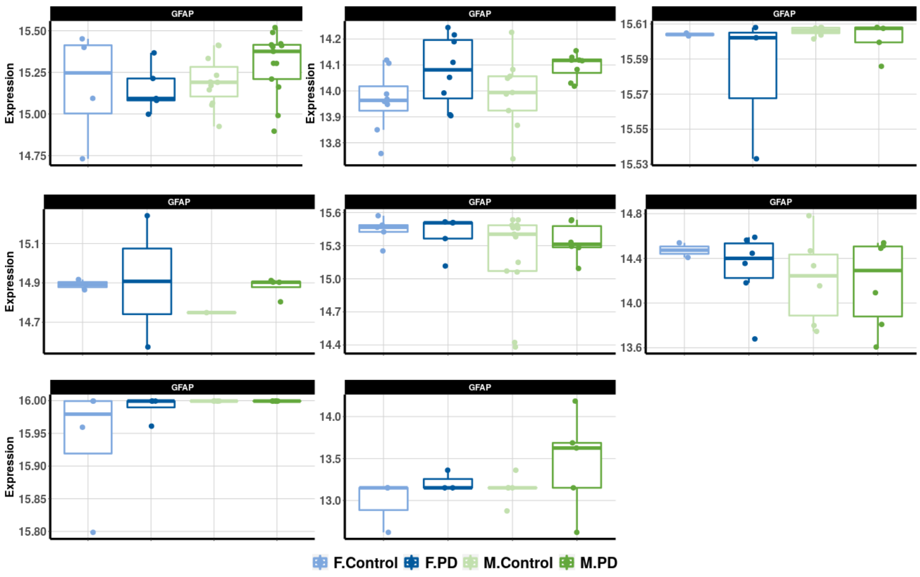

Supplementary Figure 3: Expression levels of GFAP (astrocytes). Expression levels in the bulk transcriptomics datasets used in the *substantia nigra* meta-analysis. Each dataset is represented by four boxplots representing the four patient categories of interest (female controls, female patients, male controls and male patients). Datasets from left to right and from top to bottom: GSE8397, GSE20159, GSE20163, GSE20164, GSE20292, GSE26927, GSE49036 and NBB. Each boxplot is represented by the median, two hinges (representing the first and third quartiles) and two whiskers (extending up to 1.5 * inter-quartile range from the hinges).

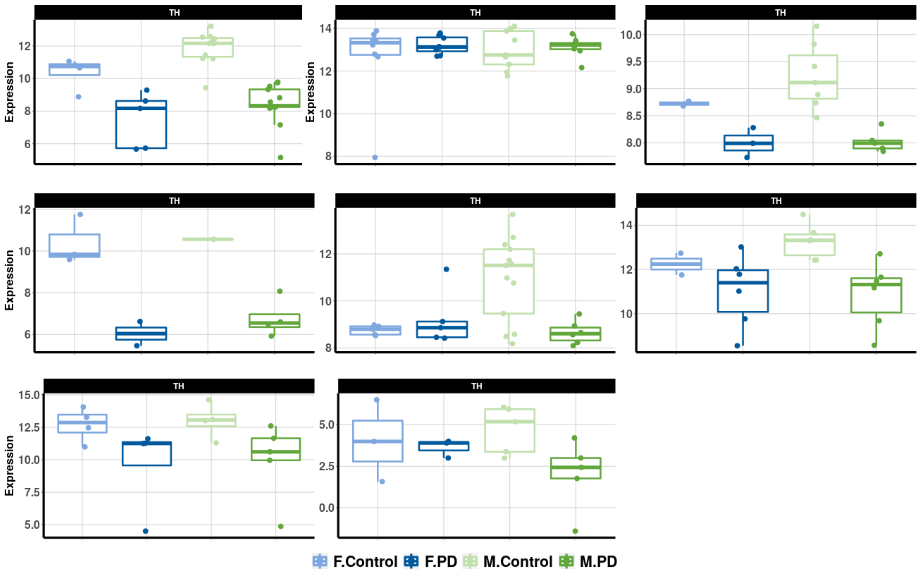

Supplementary Figure 4: Expression levels of TH (dopaminergic neurons). Expression levels in the bulk transcriptomics datasets used in the *substantia nigra* meta-analysis. Each dataset is represented by four boxplots representing the four patient categories of interest (female controls, female patients, male controls and male patients). Datasets from left to right and from top to bottom: GSE8397, GSE20159, GSE20163, GSE20164, GSE20292, GSE26927, GSE49036 and NBB. Each boxplot is represented by the median, two hinges (representing the first and third quartiles) and two whiskers (extending up to 1.5 * inter-quartile range from the hinges).

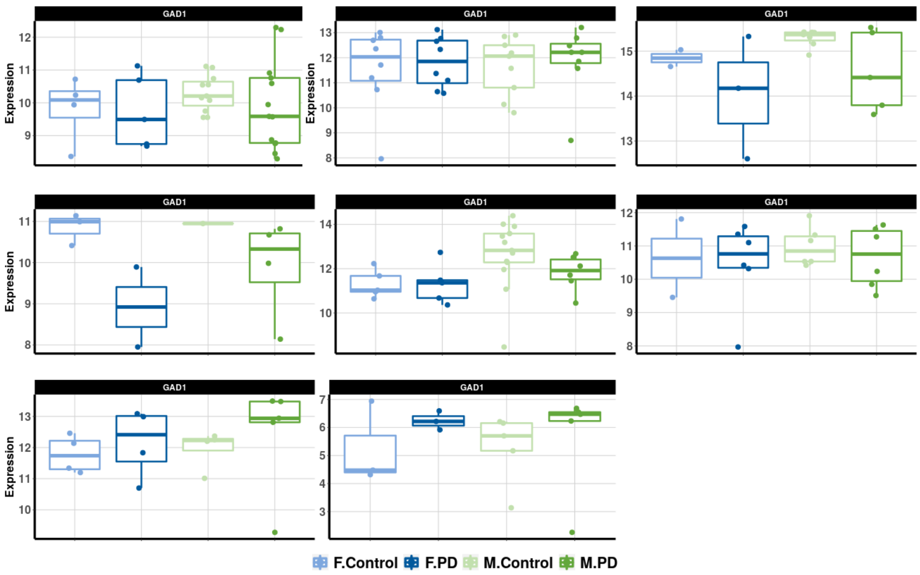

Supplementary Figure 5: Expression levels of GAD1 (GABAergic neurons). Expression levels in the bulk transcriptomics datasets used in the *substantia nigra* meta-analysis. Each dataset is represented by four boxplots representing the four patient categories of interest (female controls, female patients, male controls and male patients). Datasets from left to right and from top to bottom: GSE8397, GSE20159, GSE20163, GSE20164, GSE20292, GSE26927, GSE49036 and NBB. Each boxplot is represented by the median, two hinges (representing the first and third quartiles) and two whiskers (extending up to 1.5 * inter-quartile range from the hinges).

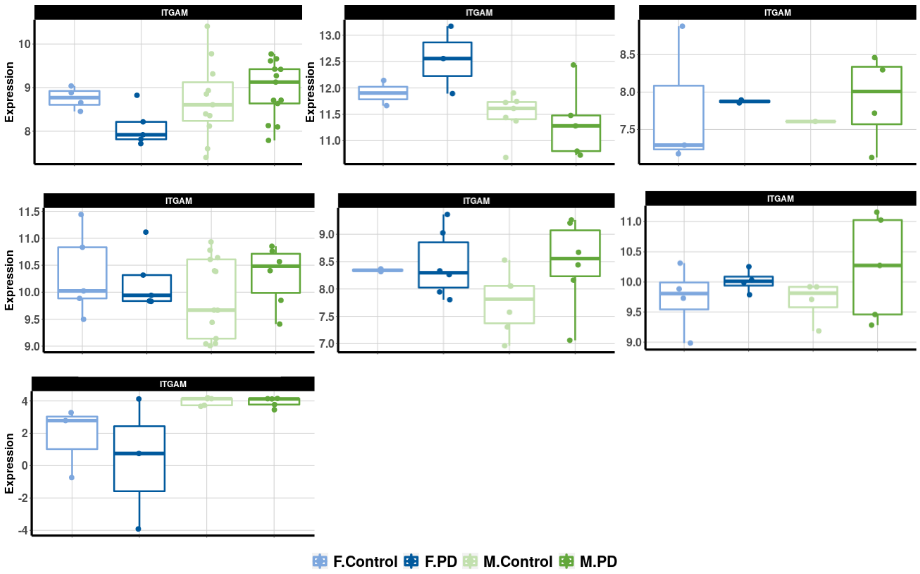

Supplementary Figure 6: Expression levels of ITGAM (microglia). Expression levels in the bulk transcriptomics datasets used in the *substantia nigra* meta-analysis. Each dataset is represented by four boxplots representing the four patient categories of interest (female controls, female patients, male controls and male patients). Datasets from left to right and from top to bottom: GSE8397, GSE20163, GSE20164, GSE20292, GSE26927, GSE49036 and NBB. Each boxplot is represented by the median, two hinges (representing the first and third quartiles) and two whiskers (extending up to 1.5 * inter-quartile range from the hinges).

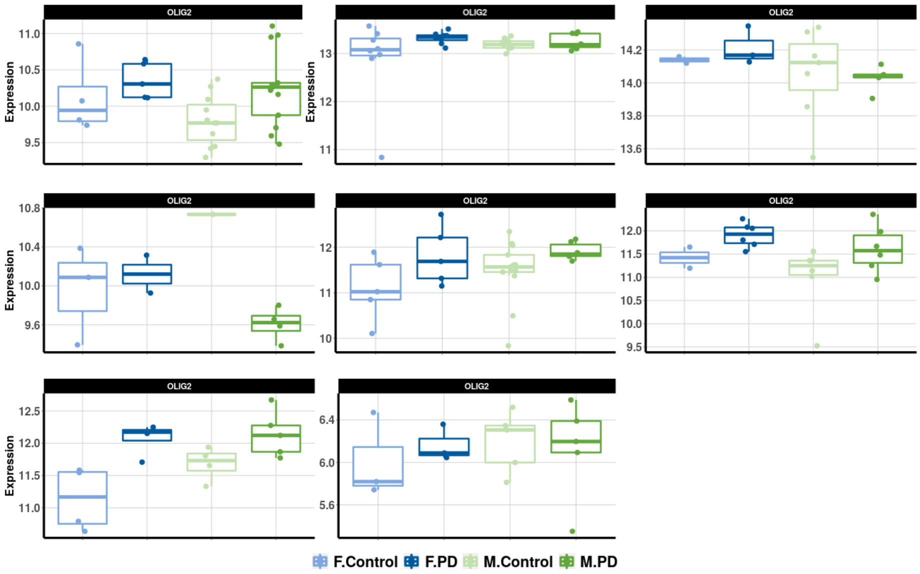

Supplementary Figure 7: Expression levels of OLIG2 (oligodendrocytes). Expression levels in the bulk transcriptomics datasets used in the *substantia nigra* meta-analysis. Each dataset is represented by four boxplots representing the four patient categories of interest (female controls, female patients, male controls and male patients). Datasets from left to right and from top to bottom: GSE8397, GSE20159, GSE20163, GSE20164, GSE20292, GSE26927, GSE49036 and NBB. Each boxplot is represented by the median, two hinges (representing the first and third quartiles) and two whiskers (extending up to 1.5 * inter-quartile range from the hinges).

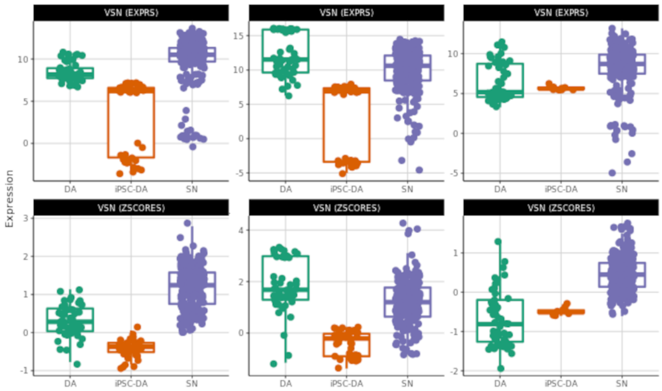

Supplementary Figure 8: Expression levels of cell type biomarkers. The expression levels are summarized across datasets targeting distinct tissues (DA in green, iPSC-DA in orange and SN in purple). (Left) Astrocyte biomarker *ALDH1L1*. (Center) Dopaminergic neuron biomarker *SLC6A3*. (Right) Microglia biomarker *ITGAM*. For each gene, either the pre-processed expression levels (top) or the associated Z-scores (bottom) are used to compare expression levels across datasets. Each boxplot is represented by the median, two hinges (representing the first and third quartiles) and two whiskers (extending up to 1.5 * inter-quartile range from the hinges).

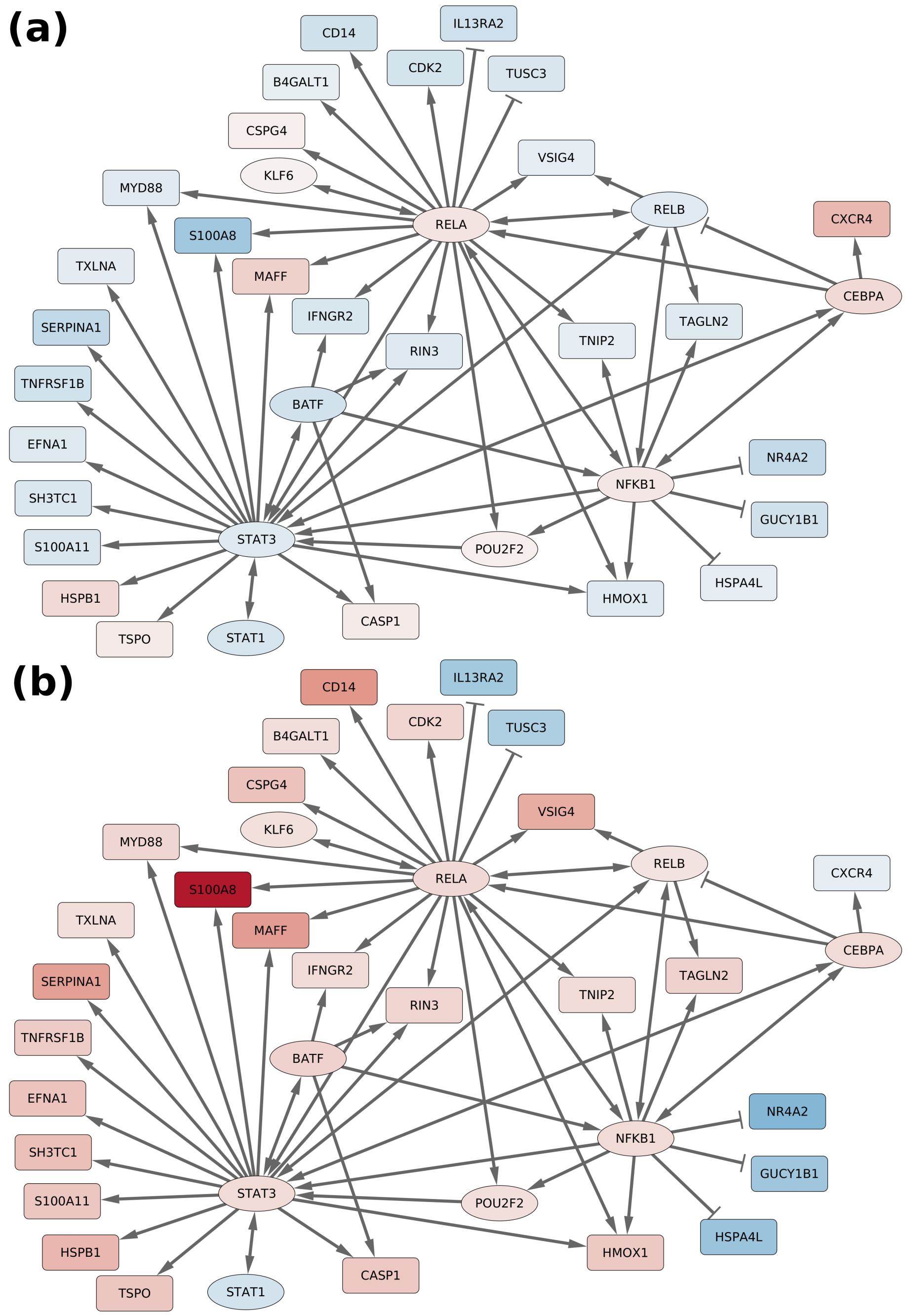

Supplementary Figure 9: Enlarged visualization of the regulatory network. This graph represents an enlarged visualization of the regulatory sub-network centered around selected transcription factors and presented in main Figure 4. The transcription factors are selected depending on the over-representation of their known targets within the differentially expressed genes, favoring the factors whose enrichment differs between the female and male analyses. Only the target genes for which the lowest FDR (across the male and female analyses) was below 0.05 are included (FDR = 0.01 for the networks presented in Figure 4). Both networks share the same content and layout but the nodes are colored according to a blue to red color gradient reflecting their cross-study log. fold change for the female (A) and male (B) analyses. The selected transcription factors are represented as ellipses, whereas other genes are represented as boxes.

References

[1] Marzia Baldereschi et al. 'Parkinson's disease and parkinsonism in a longitudinal study: Two-fold higher incidence in men'. In: *Neurology* 55.9 (2000), pp. 1358–1363. ISSN: 00283878.

[2] Luis Erik Clavería et al. 'Prevalence of Parkinson's Disease in Cantalejo, Spain: A Door-to-Door Survey'. eng. In: *Mov Disord* 17.2 (Mar. 2002), pp. 242–249. ISSN: 0885-3185. DOI: 10.1002/mds.10087.

[3] Julián Benito-León et al. 'Prevalence of PD and other types of parkinsonism in three elderly populations of central Spain'. In: *Movement Disorders* 18.3 (2003), pp. 267–274. ISSN: 08853185.

[4] Stephen K. Van Den Eeden et al. 'Incidence of Parkinson's disease: Variation by age, gender, and race/ethnicity'. In: *American Journal of Epidemiology* 157.11 (2003), pp. 1015–1022. ISSN: 00029262.

[5] L. M.L. De Lau et al. 'Incidence of parkinsonism and Parkinson disease in a general population: The Rotterdam Study'. In: *Neurology* 63.7 (2004), pp. 1240–1244. ISSN: 00283878.

[6] G. F. Wooten, L. J. Currie, V. E. Bovbjerg, J. K. Lee and J. Patrie. 'Are men at greater risk for Parkinson's disease than women?' In: *Journal of Neurology, Neurosurgery and Psychiatry* 75.4 (2004), pp. 637–639. ISSN: 00223050.

[7] Yi-Chieh Chen et al. 'Nonmotor Symptoms of 820 Taiwanese Patients with Parkinson's Disease: An Exploratory-Comparative Study'. en. In: *J Neurol* 267.5 (May 2020), pp. 1499–1507. ISSN: 1432-1459. DOI: 10.1007/s00415-020-09708-4.

[8] Ga Eun Nam et al. 'Metabolic Syndrome and Risk of Parkinson Disease: A Nationwide Cohort Study'. en. In: *PLOS Medicine* 15.8 (Aug. 2018), e1002640. ISSN: 1549-1676. DOI: 10.1371/journal.pmed.1002640.

[9] D. Georgiev, K. Hamberg, M. Hariz, L. Forsgren and G.-M. Hariz. 'Gender Differences in Parkinson's Disease: A Clinical Perspective'. en. In: *Acta Neurologica Scandinavica* 136.6 (2017), pp. 570–584. ISSN: 1600-0404. DOI: 10.1111/ane.12796.

[10] Chun-lin Ma et al. 'The Prevalence and Incidence of Parkinson's Disease in China: A Systematic Review and Meta-Analysis'. en. In: *J Neural Transm* 121.2 (Feb. 2014), pp. 123–134. ISSN: 1435-1463. DOI: 10.1007/s00702-013-1092-z.

[11] Yang Song, Zhuqin Gu, Jing An, Piu Chan and Chinese Parkinson Study Group. 'Gender Differences on Motor and Non-Motor Symptoms of de Novo Patients with Early Parkinson's Disease'. en. In: *Neurol Sci* 35.12 (Dec. 2014), pp. 1991–1996. ISSN: 1590-3478. DOI: 10.1007/s10072-014-1879-1.

[12] K. S. M. Taylor, J. A. Cook and C. E. Counsell. 'Heterogeneity in Male to Female Risk for Parkinson's Disease'. en. In: *Journal of Neurology, Neurosurgery & Psychiatry* 78.8 (Aug. 2007), pp. 905–906. ISSN: 0022-3050, 1468-330X. DOI: 10.1136/jnnp.2006.104695.

[13] Lei Cui et al. 'Prevalence of Alzheimer's Disease and Parkinson's Disease in China: An Updated Systematical Analysis'. English. In: *Front. Aging Neurosci.* 12 (2020). ISSN: 1663-4365. DOI: 10.3389/fnagi.2020.603854.

[14] Tamara Pringsheim, Nathalie Jette, Alexandra Frolkis and Thomas D. L. Steeves. 'The Prevalence of Parkinson's Disease: A Systematic Review and Meta-Analysis'. en. In: *Movement Disorders* 29.13 (2014), pp. 1583–1590. ISSN: 1531-8257. DOI: 10.1002/mds.25945.

[15] THE GTEX CONSORTIUM. 'The GTEx Consortium Atlas of Genetic Regulatory Effects across Human Tissues'. In: *Science* 369.6509 (Sept. 2020), pp. 1318–1330. DOI: 10.1126/science.aaz1776.

[16] Mike A. Nalls et al. 'Large-Scale Meta-Analysis of Genome-Wide Association Data Identifies Six New Risk Loci for Parkinson's Disease'. In: *Nat Genet* 46.9 (Sept. 2014), pp. 989–993. ISSN: 1546-1718. DOI: 10.1038/ng.3043.

[17] Diana Chang et al. 'A Meta-Analysis of Genome-Wide Association Studies Identifies 17 New Parkinson's Disease Risk Loci'. In: *Nat Genet* 49.10 (Oct. 2017), pp. 1511–1516. ISSN: 1546-1718. DOI: 10.1038/ng.3955.

[18] Mike A. Nalls et al. 'Identification of Novel Risk Loci, Causal Insights, and Heritable Risk for Parkinson's Disease: A Meta-Analysis of Genome-Wide Association Studies'. In: *Lancet Neurol* 18.12 (Dec. 2019), pp. 1091–1102. ISSN: 1474-4465. DOI: 10.1016/S1474-4422(19)30320-5.

[19] Amit Sharma et al. 'Advanced Glycation End Products and Protein Carbonyl Levels in Plasma Reveal Sex-Specific Differences in Parkinson's and Alzheimer's Disease'. en. In: *Redox Biology* 34 (July 2020), p. 101546. ISSN: 2213-2317. DOI: 10.1016/j.redox.2020.101546.

[20] Nobuyuki Sasaki et al. 'Advanced Glycation End Products in Alzheimer's Disease and Other Neurodegenerative Diseases'. en. In: *The American Journal of Pathology* 153.4 (Oct. 1998), pp. 1149–1155. ISSN: 0002-9440. DOI: 10.1016/S0002-9440(10)65659-3.

[21] Juan J. García-Vallejo, Sonja I. Gringhuis, Willem van Dijk and Irma van Die. 'Gene Expression Analysis of Glycosylation-Related Genes by Real-Time Polymerase Chain Reaction'. eng. In: *Methods Mol Biol* 347 (2006), pp. 187–209. ISSN: 1064-3745. DOI: 10.1385/1-59745-167-3:187.

[22] Joshua D. Welch et al. 'Single-Cell Multi-Omic Integration Compares and Contrasts Features of Brain Cell Identity'. eng. In: *Cell* 177.7 (June 2019), 1873–1887.e17. ISSN: 1097-4172. DOI: 10.1016/j.cell.2019.05.006.

[23] Regina H. Reynolds et al. 'Moving beyond Neurons: The Role of Cell Type-Specific Gene Regulation in Parkinson's Disease Heritability'. eng. In: *NPJ Parkinsons Dis* 5 (2019), p. 6. ISSN: 2373-8057. DOI: 10.1038/s41531-019-0076-6.

[24] Arpiar Saunders et al. 'Molecular Diversity and Specializations among the Cells of the Adult Mouse Brain'. en. In: *Cell* 174.4 (Aug. 2018), 1015–1030.e16. ISSN: 0092-8674. DOI: 10.1016/j.cell.2018.07.028.

[25] Devika Agarwal et al. 'A Single-Cell Atlas of the Human Substantia Nigra Reveals Cell-Specific Pathways Associated with Neurological Disorders'. en. In: *Nature Communications* 11.1 (Aug. 2020), p. 4183. ISSN: 2041-1723. DOI: 10.1038/s41467-020-17876-0.

[26] Ippolita Cantuti-Castelvetri et al. 'Effects of Gender on Nigral Gene Expression and Parkinson Disease'. en. In: *Neurobiology of Disease* 26.3 (June 2007), pp. 606–614. ISSN: 0969-9961. DOI: 10.1016/j.nbd.2007.02.009.

[27] Filip Simunovic, Ming Yi, Yulei Wang, Robert Stephens and Kai C. Sonntag. 'Evidence for Gender-Specific Transcriptional Profiles of Nigral Dopamine Neurons in Parkinson Disease'. eng. In: *PLoS One* 5.1 (Jan. 2010), e8856. ISSN: 1932-6203. DOI: 10.1371/journal.pone.0008856.

[28] R. Ayana, Shailja Singh and Soumya Pati. 'Deconvolution of Human Brain Cell Type Transcriptomes Unraveled Microglia-Specific Potential Biomarkers'. In: *Front Neurol* 9 (Apr. 2018), p. 266. ISSN: 1664-2295. DOI: 10.3389/fneur.2018.00266.

[29] John D. Cahoy et al. 'A Transcriptome Database for Astrocytes, Neurons, and Oligodendrocytes: A New Resource for Understanding Brain Development and Function'. In: *J. Neurosci.* 28.1 (Jan. 2008), pp. 264–278. ISSN: 0270-6474, 1529-2401. DOI: 10.1523/JNEUROSCI.4178-07.2008.

[30] Elizabeth P. Crowe et al. 'Changes in the Transcriptome of Human Astrocytes Accompanying Oxidative Stress-Induced Senescence'. In: *Frontiers in Aging Neuroscience* 8 (2016), p. 208. ISSN: 1663-4365. DOI: 10.3389/fnagi.2016.00208.

[31] Jason M. Keil, Adel Qalieh and Kenneth Y. Kwan. 'Brain Transcriptome Databases: A User's Guide'. In: *J. Neurosci.* 38.10 (Mar. 2018), pp. 2399–2412. ISSN: 0270-6474, 1529-2401. DOI: 10.1523/JNEUROSCI.1930-17.2018.

[32] Rhonda R. Voskuhl et al. 'Gene Expression in Oligodendrocytes during Remyelination Reveals Cholesterol Homeostasis as a Therapeutic Target in Multiple Sclerosis'. In: *PNAS* 116.20 (May 2019), pp. 10130–10139. ISSN: 0027-8424, 1091-6490. DOI: 10.1073/pnas.1821306116.

[33] S. Smajić et al. *Single-Cell Sequencing of the Human Midbrain Reveals Glial Activation and a Neuronal State Specific to Parkinson's Disease*. Sept. 2020. DOI: 10.1101/2020.09.28.20202812.

[34] Gonzalo S. Nido et al. 'Common Gene Expression Signatures in Parkinson's Disease Are Driven by Changes in Cell Composition'. In: *Acta Neuropathologica Communications* 8.1 (Apr. 2020), p. 55. ISSN: 2051-5960. DOI: 10.1186/s40478-020-00932-7.

[35] Aaron M. Newman et al. 'Determining Cell Type Abundance and Expression from Bulk Tissues with Digital Cytometry'. In: *Nat Biotechnol* 37.7 (July 2019), pp. 773–782. ISSN: 1546-1696. DOI: 10.1038/s41587-019-0114-2.
