# Supplementary material for: Systems level analysis of sex-dependent gene expression changes in Parkinson’s disease
Source: NPJ Parkinsons Dis. 2023 Jan 21;9:8. doi: 10.1038/s41531-023-00446-8 (PMC9867746; doi:10.1038/s41531-023-00446-8)
Supplement: Supplementary file 2 — Reporting Summary [file 41531_2023_446_MOESM2_ESM.pdf]

nature portfolio

Corresponding author(s): Enrico Glaab
Last updated by author(s): Dec 16, 2022

Reporting Summary

Nature Portfolio wishes to improve the reproducibility of the work that we publish. This form provides structure for consistency and transparency in reporting. For further information on Nature Portfolio policies, see our Editorial Policies and the Editorial Policy Checklist.

Statistics

For all statistical analyses, confirm that the following items are present in the figure legend, table legend, main text, or Methods section.

n/a	Confirmed	
☐	☒	The exact sample size (*n*) for each experimental group/condition, given as a discrete number and unit of measurement
☐	☒	A statement on whether measurements were taken from distinct samples or whether the same sample was measured repeatedly
☐	☒	The statistical test(s) used AND whether they are one- or two-sided<br>*Only common tests should be described solely by name; describe more complex techniques in the Methods section.*
☐	☒	A description of all covariates tested
☐	☒	A description of any assumptions or corrections, such as tests of normality and adjustment for multiple comparisons
☒	☐	A full description of the statistical parameters including central tendency (e.g. means) or other basic estimates (e.g. regression coefficient) AND variation (e.g. standard deviation) or associated estimates of uncertainty (e.g. confidence intervals)
☒	☐	For null hypothesis testing, the test statistic (e.g. *F*, *t*, *r*) with confidence intervals, effect sizes, degrees of freedom and *P* value noted<br>*Give P values as exact values whenever suitable.*
☒	☐	For Bayesian analysis, information on the choice of priors and Markov chain Monte Carlo settings
☒	☐	For hierarchical and complex designs, identification of the appropriate level for tests and full reporting of outcomes
☒	☐	Estimates of effect sizes (e.g. Cohen's *d*, Pearson's *r*), indicating how they were calculated

Our web collection on statistics for biologists contains articles on many of the points above.

Software and code

Policy information about availability of computer code

Data collection	No software was used for data collection.
Data analysis	The code used for the analyses described in this article is shared on gitlab (https://gitlab.lcsb.uni.lu/bds/geneder/geneder_core).

For manuscripts utilizing custom algorithms or software that are central to the research but not yet described in published literature, software must be made available to editors and reviewers. We strongly encourage code deposition in a community repository (e.g. GitHub). See the Nature Portfolio guidelines for submitting code & software for further information.

Data

Policy information about availability of data

All manuscripts must include a data availability statement. This statement should provide the following information, where applicable:
- Accession codes, unique identifiers, or web links for publicly available datasets
- A description of any restrictions on data availability
- For clinical datasets or third party data, please ensure that the statement adheres to our policy

All data and materials associated with this publication, including links to the data as well as all the supplementary files mentioned in the manuscript are hosted on a dedicated webpage (https://doi.org/10.17881/hpbx-y095). For the RNA-seq data measured in-house, the post-mortem human brain samples were obtained from The Netherlands Brain Bank, Netherlands Institute for Neuroscience (Amsterdam, The Netherlands; open access: http://www.brainbank.nl). All material has been collected from donors for or from whom a written informed consent for a brain autopsy and the use of the material and clinical information for research purposes

had been obtained by the NBB. The data can be accessed on the Gene Expression Omnibus database under the identifier GSE168496 (https://www.ncbi.nlm.nih.gov/geo/query/acc.cgi?acc=GSE168496).

Human research participants

Policy information about studies involving human research participants and Sex and Gender in Research.

Reporting on sex and gender	The objective of this project is to study the differences between males and females. Therefore, sex-specific analyses have been performed.
Population characteristics	20 datasets, 428 samples.<br>Disease status: 209 healthy controls and 219 Parkinson's disease patients.<br>Biological sex: 174 females, 247 males, 5 unknown sex.<br>Age: mean = 75.1 +- 11.2 years
Recruitment	No recruitment took place in the context of that study.
Ethics oversight	No ethics approval was necessary for this project.

Note that full information on the approval of the study protocol must also be provided in the manuscript.

Field-specific reporting

Please select the one below that is the best fit for your research. If you are not sure, read the appropriate sections before making your selection.

☒ Life sciences ☐ Behavioural & social sciences ☐ Ecological, evolutionary & environmental sciences

For a reference copy of the document with all sections, see nature.com/documents/nr-reporting-summary-flat.pdf

Life sciences study design

All studies must disclose on these points even when the disclosure is negative.

Sample size	No sample size calculation was performed. To our knowledge, we have gathered all publicly available transcriptomics datasets profiling post-mortem substantia nigra tissues of Parkinson's disease patients.
Data exclusions	Samples were removed when:<br>- they were duplicates (same human brain, different samples across different datasets because our meta-analysis assume dataset independence)<br>- they did not pass our internal quality controls<br>- they could not be associated with a male or female sex<br>- they could not be associated with a age value<br>- they were associated with a dataset that was too small to be included in the meta-analysis
Replication	A replication of the results obtained with the bulk transcriptomics meta-analysis was performed using single-cell transcriptomics datasets.
Randomization	The aim of the project is to study molecular differences between males and females. We do not study the impact of any treatment. Therefore, randomization is not relevant for this study.
Blinding	The aim of the project is to study molecular differences between males and females. We do not study the impact of any treatment. Therefore, blinding is not relevant for this study.

Reporting for specific materials, systems and methods

We require information from authors about some types of materials, experimental systems and methods used in many studies. Here, indicate whether each material, system or method listed is relevant to your study. If you are not sure if a list item applies to your research, read the appropriate section before selecting a response.

Materials & experimental systems

n/a	Involved in the study
☒	☐ Antibodies
☒	☐ Eukaryotic cell lines
☒	☐ Palaeontology and archaeology
☒	☐ Animals and other organisms
☒	☐ Clinical data
☒	☐ Dual use research of concern

Methods

n/a	Involved in the study
☒	☐ ChIP-seq
☒	☐ Flow cytometry
☒	☐ MRI-based neuroimaging
